# Neuronal nuclear calcium signaling suppression of microglial reactivity is mediated by osteoprotegerin after traumatic brain injury

**DOI:** 10.1186/s12974-022-02634-4

**Published:** 2022-11-19

**Authors:** Albrecht Fröhlich, Florian Olde Heuvel, Rida Rehman, Sruthi Sankari Krishnamurthy, Shun Li, Zhenghui Li, David Bayer, Alison Conquest, Anna M. Hagenston, Albert Ludolph, Markus Huber-Lang, Tobias Boeckers, Bernd Knöll, Maria Cristina Morganti-Kossmann, Hilmar Bading, Francesco Roselli

**Affiliations:** 1 Dept. of Neurology, Ulm University, Ulm, Germany; 2 CEMMA (Cellular and Molecular Mechanisms in Aging) Research Training Group, Ulm, Germany; 3 Dept. of Neurosurgery, Kaifeng Central Hospital, Kaifeng, China; 4 National Trauma Research Institute and Department of Neurosurgery, The Alfred Hospital, Melbourne, Australia; 5 Interdisciplinary Center for Neurosciences, Department of Neurobiology, Heidelberg University, Heidelberg, Germany; 6 German Center for Neurodegenerative Diseases (DZNE)-Ulm, Ulm, Germany; 7 Institute for Clinical and Experimental Trauma Immunology, Ulm University, Ulm, Germany; 8 Institute for Anatomy and Cell Biology, Ulm University, Ulm, Germany; 9 Institute of Neurobiochemistry, Ulm University, Ulm, Germany; 10 Department of Child Health, Barrow Neurological Institute at Phoenix Children’s Hospital, University of Arizona College of Medicine, Phoenix, Phoenix, AZ USA; 11 Present Address: Center for Biomedical Research, Helmholtzstrasse 8, 89081 Ulm, Germany

**Keywords:** Traumatic brain injury, Microglia, Nuclear calcium, Osteoprotegerin, Synapses

## Abstract

**Background:**

Traumatic brain injury (TBI) is characterized by massive changes in neuronal excitation, from acute excitotoxicity to chronic hyper- or hypoexcitability. Nuclear calcium signaling pathways are involved in translating changes in synaptic inputs and neuronal activity into discrete transcriptional programs which not only affect neuronal survival and synaptic integrity, but also the crosstalk between neurons and glial cells. Here, we report the effects of blunting neuronal nuclear calcium signals in the context of TBI.

**Methods:**

We used AAV vectors to express the genetically encoded and nuclear-targeted calcium buffer parvalbumin (PV.NLS.mCherry) or the calcium/calmodulin buffer CaMBP4.mCherry in neurons only. Upon TBI, the extent of neuroinflammation, neuronal death and synaptic loss were assessed by immunohistochemistry and targeted transcriptome analysis. Modulation of the overall level of neuronal activity was achieved by PSAM/PSEM chemogenetics targeted to parvalbumin interneurons. The functional impact of neuronal nuclear calcium buffering in TBI was assessed by quantification of spontaneous whisking.

**Results:**

Buffering neuronal nuclear calcium unexpectedly resulted in a massive and long-lasting increase in the recruitment of reactive microglia to the injury site, which was characterized by a disease-associated and phagocytic phenotype. This effect was accompanied by a substantial surge in synaptic loss and significantly reduced whisking activity. Transcriptome analysis revealed a complex effect of TBI in the context of neuronal nuclear calcium buffering, with upregulation of complement factors, chemokines and interferon-response genes, as well as the downregulation of synaptic genes and epigenetic regulators compared to control conditions. Notably, nuclear calcium buffering led to a substantial loss in neuronal osteoprotegerin (OPG), whereas stimulation of neuronal firing induced OPG expression. Viral re-expression of OPG resulted in decreased microglial recruitment and synaptic loss. OPG upregulation was also observed in the CSF of human TBI patients, underscoring its translational value.

**Conclusion:**

Neuronal nuclear calcium signals regulate the degree of microglial recruitment and reactivity upon TBI via, among others, osteoprotegerin signals. Our findings support a model whereby neuronal activity altered after TBI exerts a powerful impact on the neuroinflammatory cascade, which in turn contributes to the overall loss of synapses and functional impairment.

**Supplementary Information:**

The online version contains supplementary material available at 10.1186/s12974-022-02634-4.

## Introduction

Traumatic brain injury (TBI) is characterized by the unfolding of multiple pathogenic cascades involving neurons, astrocytes, microglia, and several subpopulations of infiltrating immune cells [47]. The overall neuroinflammatory and neuroimmune response is shaped by the interactions among multiple subpopulations of immune cells but also by their relationship with glial cells and with the neurons themselves [51].

Neuronal activity is a critical factor in shaping the vulnerability of neurons in TBI. Neuronal hyperexcitation is thought to substantially contribute, through excitotoxicity, to neuronal loss (secondary injury) occurring minutes-to-hours after the primary lesion [53, 64, 78]. In the excitotoxic phase, cytoplasmic Ca^2+^ overload impairs mitochondrial metabolism [58], drives the calpain-mediated disassembly of the cytoskeleton [45] and suppresses transcriptional responses [6, 31]. At later stages (several hours-to-days following brain injury), electrophysiological recordings have shown hypoexcitation of cortical neurons [2, 35] and at these timepoints stimulation of neuronal activity by glutamatergic agonists [53], chemogenetics [14, 15] and GABA receptor blockers [84] is neuroprotective.

Interestingly, neuronal activity does not only affect neuronal vulnerability, but it has been shown to affect microglial transcriptome [4] and neuroinflammatory responses. Increased neuronal excitation (as in epileptic seizures, removal of extracellular Calcium or chemogenetic stimulation [21, 67], enhances the extension of processes and the convergent recruitment of microglia through adenosine signaling [23] as well as through fractalkine and IL-1β release [22]. Notably, also the decrease in neuronal activity substantially affects microglial reactivity: chemogenetic suppression of neuronal firing upregulates the extension of microglial processes [67] and global decrease in neuronal network activity appears to promote microglial process surveillance through noradrenergic signaling [41, 65]. Moreover, deletion of activity-dependent transcription factor ATF3 in neurons results in a substantial increase in neuroinflammation upon TBI [25]. Thus, the extreme changes in neuronal excitation and activity occurring in TBI may be well posited to affect microglial dynamics occurring in this condition [34, 47, 60].

The extent and pattern of neuronal firing is translated into transcriptional programs through changes in nuclear calcium (NC) signaling [5, 12]. Upon neuronal depolarization [80], increased levels of cytoplasmic calcium (Ca^2+^) evoke the elevation of NC, which, in turn, activates nuclearly localized Ca^2+^/calmodulin-dependent kinases including CamKIV as well as Ca^2+^-sensitive TF such as DREAM [5, 48]. NC drives the transcription of a substantial number of genes associated with the preservation of structural integrity of neurons [44] and with anti-apoptotic responses [1, 19] Blunting NC has been previously achieved by overexpression of the high-affinity Ca^2+^-binding protein parvalbumin in the nucleus, through a strong nuclear localization Signal (PV.NLS construct; [55]. Alternatively, suppression of NC signaling has also been accomplished with the sequestration of nuclear Ca^2+^/calmodulin complex by an engineered Ca^2+^/CaM buffer (CaMBP4; [73]). Both PV.NLS and nuclear CaMBP4 have been shown to strongly downregulate transcriptional responses associated with synaptic activity and NC [7, 54, 62] and to regulate the morphology of dendritic arborizations [44]. However, the role of NC signaling in the crosstalk between neurons and microglia has not been investigated before.

In the present study, we found that suppressing neuronal NC results, upon TBI, in a massive build-up of reactive microglia with disease-associated-like phenotypes (Disease-Associated Microglia, as defined by [36]. Notably, the comparison of transcriptomes revealed a distinct loss of osteoprotegerin (OPG) upon NC blunting, whereas microglial accumulation and synaptic loss were reversed by the neuronal re-expression of OPG. Thus, OPG emerges as a new NC-dependent mediator of neuron–microglia interactions in the neuroinflammatory response following TBI.

## Results

### Buffering nuclear calcium in neurons enhances the early accumulation of microglia upon TBI

In order to demonstrate the effective target engagement of PV.NLS expressed in neurons, we assessed the levels of phospho-CREB (pCREB) 3 h after TBI (since NC is critical for the phosphorylation of CREB; [8, 30, 40].

**Fig. 1:**
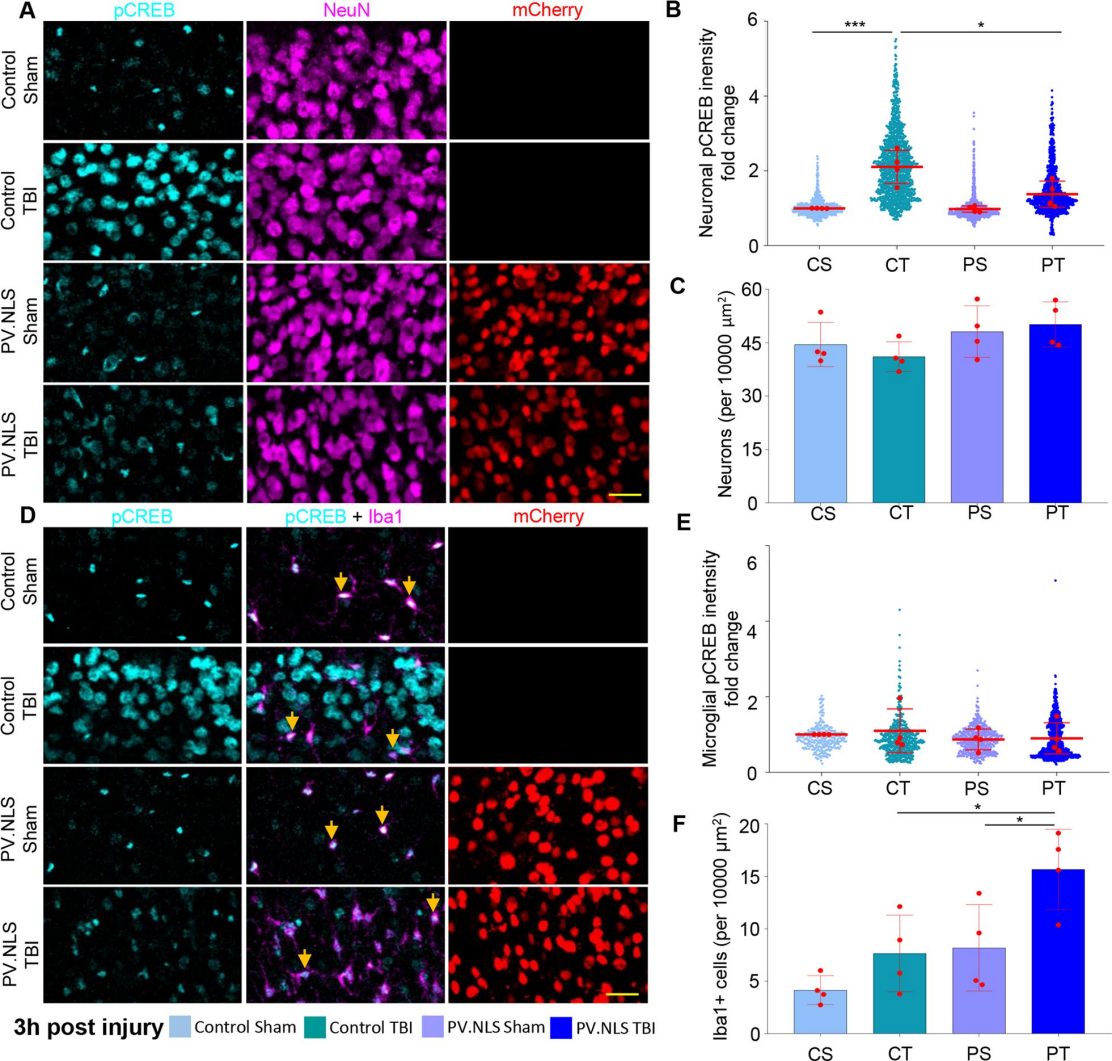
Buffering nuclear calcium in neurons enhances the accumulation of microglia 3 h after TBI. **A**, **B** Significant increase of neuronal pCREB intensity 3 h post-TBI compared to Sham (CS vs CT; 1.00 ± 0.01 vs 2.11 ± 0.44). Buffering of nuclear Calcium significantly decreases neuronal pCREB intensity 3 h post-TBI (CT vs PT; 2.11 ± 0.44 vs 1.37 ± 0.35). Red datapoints indicate average per animal (*N* = 4, used for statistics); smaller datapoints depict individual neurons. Mean ± SD. **C** Neuronal density at the injury site 3 h post-TBI; no difference across groups. *N* = 4. **D**, **E** Unchanged microglial pCREB intensity 3 h post-TBI compared to sham. Buffering of nuclear calcium does not significantly increase microglial pCREB intensity 3 h post-TBI. *N* = 4. **F** Buffering of nuclear Calcium significantly increased microglia density upon TBI (CT vs PT; 7.65 ± 3.65 vs 15.66 ± 3.81). No significant difference in microglia density in animals injected with control virus (CS vs CT). Data are shown as mean ± SD. *N* = 4 mice. Scale bar: 25 µm. *p < 0.05; ***p < 0.001

AAV9 encoding for hSyn::PV.NLS-mCherry (or an empty vector for control) was injected into the somatosensory cortex of adult mice, generating > 90% infection efficiency (30 days after injection > 90% of NeuN + cells were mCherry + (Additional file 1: Fig. S1A, B). Thirty days after AAV9 injection, mice were randomized to undergo either mild TBI (all animals scored 0 at the NSS test; scores of individual animals are reported in Additional file 6: Table S1) or sham surgery. There were a total of four experimental groups: 1. empty vector (control) sham (CS); 2. control TBI (CT); 3. PV.NLS sham (PS); 4. PV.NLS TBI (PT), all of which were killed 3 h after treatment. CT samples displayed a significant increase in neuronal pCREB in the site of injury compared to CS (as previously reported; [14], but the expression of PV.NLS largely blunted it (Fig. 1A, B). At this time point no neuronal loss was detected, irrespective of PV.NLS expression (Fig. 1C. Notably, a large number of small, elongated pCREB + nuclei were found in PT, but not in CT images; co-immunostaining of pCREB with GFAP and IBA1 revealed that almost all the pCREB + , small, elongated nuclei were detected in IBA1 + cells and therefore identified as microglia (> 98%; (Additional file 1: Fig. S1C, D)).

In an independent set of experiments, we explored the effect of neuronal NC blunting on microglia upon TBI. In agreement with the spatially heterogeneous nature of TBI lesions at this time point [14, 15], we considered two regions of interest: one located at the center of the lesion (“core”) and the other located at a fixed lateral distance from the core (“perilesional area”, Additional file 2: Fig. S2A). At 3 h post-TBI, CT samples displayed only a small increase in IBA1 + cells compared to CS, whereas PT samples showed a massive increase in the number of IBA1 + cells in the site of injury (Fig. 1D, F) as well as in the perilesional area (Additional file 1: Fig. S1E, F). pCREB levels in microglia were not significantly altered across experimental groups (Fig. 1D, E). Thus, buffering of neuronal NC in the acute phases of TBI unexpectedly resulted in the massive increase in local microglia.

### Buffering of neuronal nuclear calcium induces a disease-associated microglia (DAM)-like phenotype upon TBI

We further characterized the IBA1 + population expanded in PT by immunostaining with the microglia marker TMEM119 and the disease-associated microglia (DAM)-like markers CD11c and CST7 [36, 57]. We also determined the expression of CD169, a marker of pathogenic phagocytes [11, 56].

**Fig. 2:**
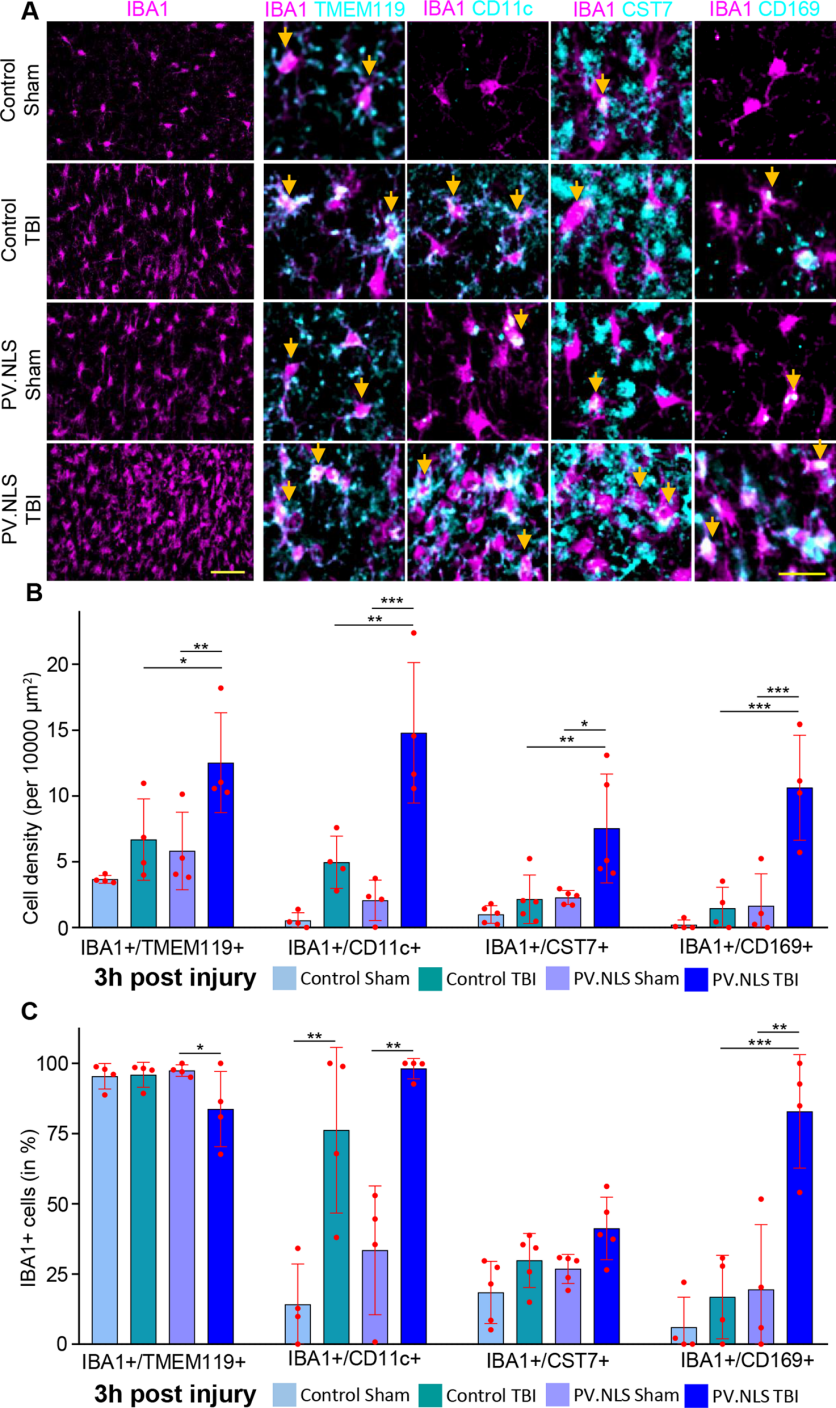
Buffering neuronal nuclear calcium induces a disease-associated microglia (DAM)-like phenotype upon TBI. **A**, **B** Nuclear calcium buffering significantly increases the density of IBA1 + /TMEM119 + , IBA1 + /CD11c + and IBA1 + /CST7 + cells 3 h post-TBI (CT vs PT; IBA1 + /TMEM119 + : 6.68 ± 3.09 vs 12.52 ± 3.79; IBA1 + /CD11c + : 4.96 ± 1.99 vs 14.80 ± 5.33; IBA1 + /CST7 + : 2.15 ± 1.84 vs 7.53 ± 4.14). Significant increase in IBA1 + /CD169 + (PS vs PT; 1.638 ± 2.446 vs 10.630 ± 3.989). *N* = 4–5. **C** Nuclear calcium buffering significantly increase the fraction of IBA1 + /CD11c (vs total IBA1 + cells) 3 h post-TBI compared to PV.NLS-Sham (CS vs CT; 14.18 ± 14.37% vs 76.19 ± 29.49%), but not to TBI alone (CT vs PT; 76.19 ± 29.49% vs 98.13 ± 3.64%). The fraction of IBA1 + /CD169 double-positive cells was increased upon TBI by nuclear calcium buffering cells (CT vs PT; 16.80 ± 14.86% vs 82.90 ± 20.20%). *N* = 4–5. Scale bar 50 µm (overview) and 25 µm (inserts). Arrows indicate double-positive cells. Data are shown as mean ± SD. *p < 0.05; **p < 0.01; ***p < 0.001

Across the four experimental groups, over > 95% of IBA1 + cells were also TMEM119 + at 3 h post-injury (Fig. 2A, C), indicating that the contribution of infiltrating peripheral cells was comparatively minimal at this time point. Interestingly, PT samples showed the highest density of TMEM119 + cells (Fig. 2B).

The density of IBA1 + CD11c + cells was massively increased in PT samples compared to either CT or PS (Fig. 2A, B); however, their portion compared to overall IBA1 + population was unchanged (Fig. 2C). Likewise, the density of IBA1 + /CST7 + cells was significantly increased in PT samples compared to CS, CT or PS but the presence of total IBA1 + cells was comparable in CT and PT. On the other hand, only very few IBA1 + cells co-expressed CD169 in CS, CT or PT samples, but the number of IBA1 + /CD169 + cells was massively increased in PT brains. Most notably, the population of IBA1 + /CD169 + cells was also substantially increased in PT, implying that the strong expression of CD169 + in microglia corresponded to a reactive phenotype following the NC blockade as well as TBI (Fig. 2A, C). Thus, the blockade of NC results, upon TBI, in the appearance of a large microglial population with a DAM-like and unique phenotype characterized by high CD169 expression.

### Blockade of neuronal nuclear calcium/calmodulin signaling recapitulates the enhanced recruitment of microglia after TBI

**Fig. 3:**
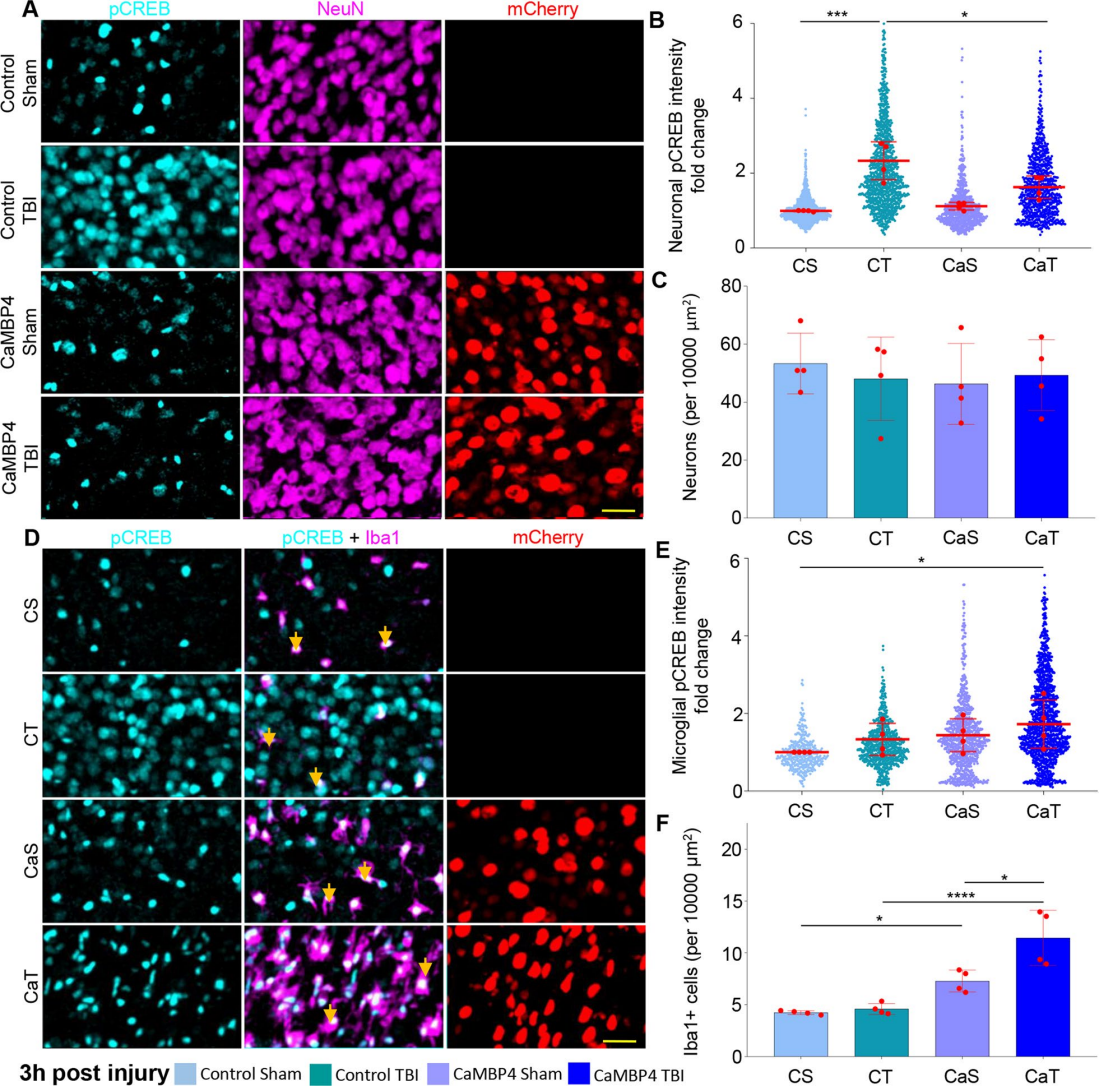
Blockade of neuronal nuclear calcium/calmodulin pathway recapitulates the enhanced recruitment of microglia after trauma. **A**, **B** Buffering of CaM by CAMBP4 significantly decreases neuronal pCREB intensity 3 h post-TBI (CT vs CaT; 2.33 ± 0.50 vs 1.62 ± 0.29). *N* = 4. *C* No significant difference of neuronal density at the injury site 3 h post-TBI. Mean ± SD. *N* = 4. **D**–**F** Buffering of CamK activation does not significantly increase microglial pCREB intensity 3 h post-TBI. **E** CT vs CaT; 1.33 ± 0.41 vs 1.72 ± 0.62; *N* = 4) but significantly increased microglia density post-TBI **F** CT vs CaT; 4.58 ± 0.53 vs 11.44 ± 2.66). Individual neurons are depicted as small datapoints; average per mouse (*N* = 4, used for statistics) depicted as red datapoints. Arrows indicate IBA1 + cells expressing pCREB. Data are shown as mean ± SD. Scale bar: 25 µm. *N* = 4. *p < 0.05; ****p < 0.0001

Since Ca^2+^/CaM-dependent kinases play a significant role in regulating signaling downstream of NC [62], we hypothesized that sequestering nuclear Ca^2+^/CaM would recapitulate the effect of blunting NC with PV.NLS. For this purpose, we expressed a CaMBP4.mCherry construct, designed to bind and sequester nuclear Ca^2+^/CaM [73] or an empty vector for control in neurons of the somatosensory cortex via AAV injection. We considered four experimental groups: 1. control sham (CS), 2. control TBI (CT); 3. CaMBP4 sham (CaS); and 4. CaMBP4 TBI (CaT). Observing the core area following TBI, the expression of CaMBP4 significantly reduced the upregulation of pCREB (Fig. 3A, B), but did not affect the vulnerability of neurons 3 h after TBI (Fig. 3C). Likewise, the expression of neuronal CaMBP4 caused a massive increase in microglial density upon TBI (Fig. 3D, F), but was ineffective in sham-treated animals. Finally, we found that, compared to CS, the levels of pCREB were substantially increased in microglial cells when CaMBP4 was expressed in neurons (Fig. 3D, E). Thus, the inhibition of CaM-dependent NC signaling exerted by CaMBP4 in neurons largely recapitulates the effects of NC buffering by PV.NLS. This data suggests that NC-dependent action on CREB phosphorylation and microglial accumulation is specific and mediated by a CaM-dependent pathway.

### Buffering of neuronal nuclear calcium enhances subacute microgliosis and synapse loss in TBI

We then explored the impact of NC buffering in TBI observed at later stages such as 24 h post-injury (1dpi) and 7d post-injury.

**Fig. 4:**
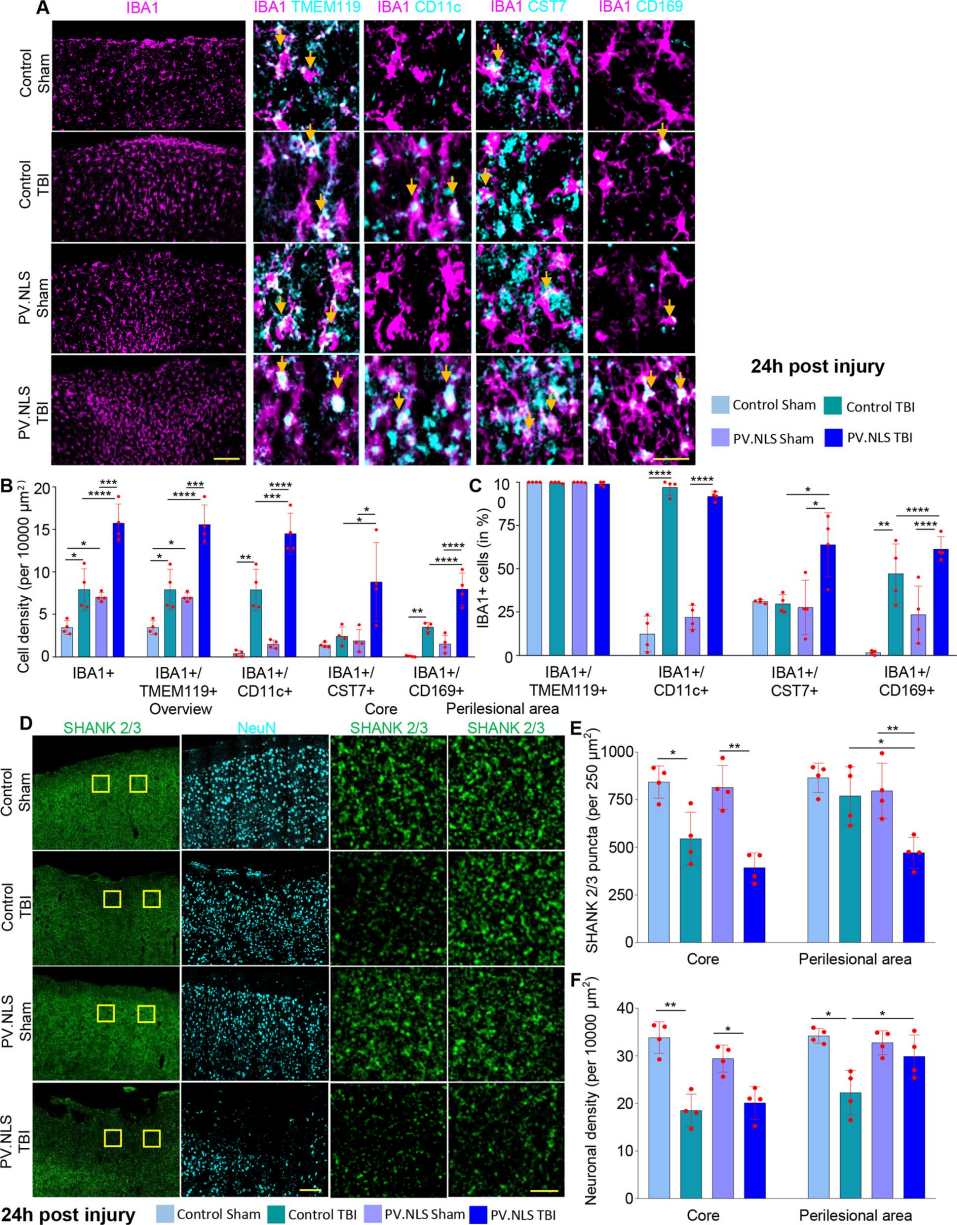
Buffering of neuronal nuclear calcium enhances subacute microgliosis and synapse loss 24 h post-trauma. **A**–**C** At 24 h after injury, neuronal nuclear Calcium buffering drives the significant increase of IBA1 + , IBA1 + /TMEM119 + , IBA1 + /CD11c + , IBA1 + /CST7 + and IBA1 + /CD169 + compared to TBI in mice injected with control AAV (**B** CT vs PT; for IBA1 + cells 7.92 ± 2.44 vs 15.74 ± 2.24; for IBA1 + /TMEM119 + cells 7.89 ± 2.40 vs 15.57 ± 2.30; for IBA1 + /CD11c + cells 7.88 ± 2.41 vs 14.50 ± 2.40; CT vs PT for IBA1 + /CST7 + cells; 2.44 ± 1.06 vs 8.79 ± 4.64; CT vs PT for IBA1 + /CD169 + cells; 3.48 ± 0.52 vs 7.95 ± 1.89). Buffering of nuclear calcium signaling in TBI did not alter the fraction of IBA1 + /CD11c + cells (**C**: CT vs PT; 97.06 ± 4.48% vs 91.80 ± 2.72%), but increased the fraction of CST7 + and CD169 +  + (CT vs PT for CST7; 29.72 ± 5.31% vs 63.82 ± 18.50%; CT vs PT vor CD169; 47.13 ± 17.14% vs 61.36 ± 7.11%). Data are shown as mean ± SD. *N* = 4. Scale bar 100 µm (overview) and 20 µm (insert). **D**–**E** TBI decreases excitatory (Shank2/3+) synaptic density in the core (CS vs CT; 843.10 ± 84.62 vs 545.32 ± 138.21) but not in the perilesional area of the injury. Buffering of nuclear calcium signaling did not worsen the core synaptic density upon TBI but significantly decreased synaptic density in the perilesional area (CT vs PT; 769.9 ± 152.8 vs 471.3 ± 80.9). *N* = 4. **F** Significant decrease in neuronal density in the injury core 24 h post-TBI (CS vs CT; 33.82 ± 3.32 vs 18.50 ± 3.44). Buffering nuclear calcium signaling did not worsen neuronal density in the core compared to TBI alone (CT vs PT; 18.50 ± 3.44 vs 20.09 ± 3.41). Significant decrease in neuronal density in the perilesional area post-TBI to sham and nuclear calcium buffering (CS vs CT; 34.2 ± 1.55 vs 22.25 ± 4.67; CT vs PT; 22.25 ± 4.67 vs 29.88 ± 4.52). Data are shown as mean ± SD. *N* = 4. Scale bar 100 µm (overview) and 5 µm (insert). *p < 0.05; **p < 0.01; ***p < 0.001; ****p < 0.0001

At 24 h post-injury, CT brains displayed an increased density of IBA1 + cells compared to sham mice (CT vs CS). However, in PT samples there was no evidence for a larger expansion of the IBA1 + population compared to CT samples, whereby cells showed a distinct ameboid morphology (Fig. 4A, B). The abundant IBA1 + population was associated with a significantly higher density (cells/area unit) of IBA1 + /TMEM119 + cells in the core and perilesional areas (Fig. 4A, B and Additional file 2: Fig. S2B-C). In addition, virtually all IBA1 + cells were TMEM119 + , indicating a limited contribution of peripheral immune cells within and around the lesion (Fig. 4C). The IBA1 + /CD11c + subpopulation was substantially larger in PT samples (Fig. 4A, B), yet maintaining a comparable component of total IBA1 + cells in PT and CT brains (Fig. 4C), suggesting an overall expansion of the microglial population in PT. Notably, the density of IBA1 + /Cst7 + as well as of IBA1 + /CD169 + cells and their relative representation was significantly increased at 24 h in PT samples (Fig. 4A-C). Thus, NC blunting resulted, upon TBI, in a substantially larger microglial population with a distinctive over-representation of CST7 + and CD169 + cells.

To assess the relationship between microglia infiltration and the extent of synapse loss, we quantified the integrity of the cortical architecture by measuring the density of excitatory synapses (number of pan-Shank + puncta per area unit), inhibitory synapses (number of Gephyrin + puncta per area unit) and the number of surviving neurons in the core and perilesional brain areas.

At 24 h after injury CT animals showed a significant loss of excitatory synapses was observed in the core of the lesion, whereas in the perilesional area the synaptic density was only slightly reduced compared to CS brains (Fig. 4D, E). In contrast, we detected a robust loss of excitatory synapses in PT samples particularly in the perilesional area, indicating that the area of synaptic involvement is much larger than expected, as clearly manifested in the low-magnification pictures (Fig. 4D). On the other hand, Gephyrin puncta (inhibitory synapses) were reduced to a similar extent in the core of the lesion as well as in the perilesional area at 24 h (Additional file 2: Fig. S2D–F).

Together, these experiments suggest that NC blockade resulted in a more extensive microgliosis and an exacerbated loss of excitatory synapses in the cortical area affected by TBI at 24 h post injury. Neuronal density was substantially decreased in the core of both CT and PT groups (Fig. 4F). Interestingly, an increased preservation of neuronal density was found in the perilesional area of PT brains despite the increased microgliosis and excitatory synaptic loss.

**Fig. 5:**
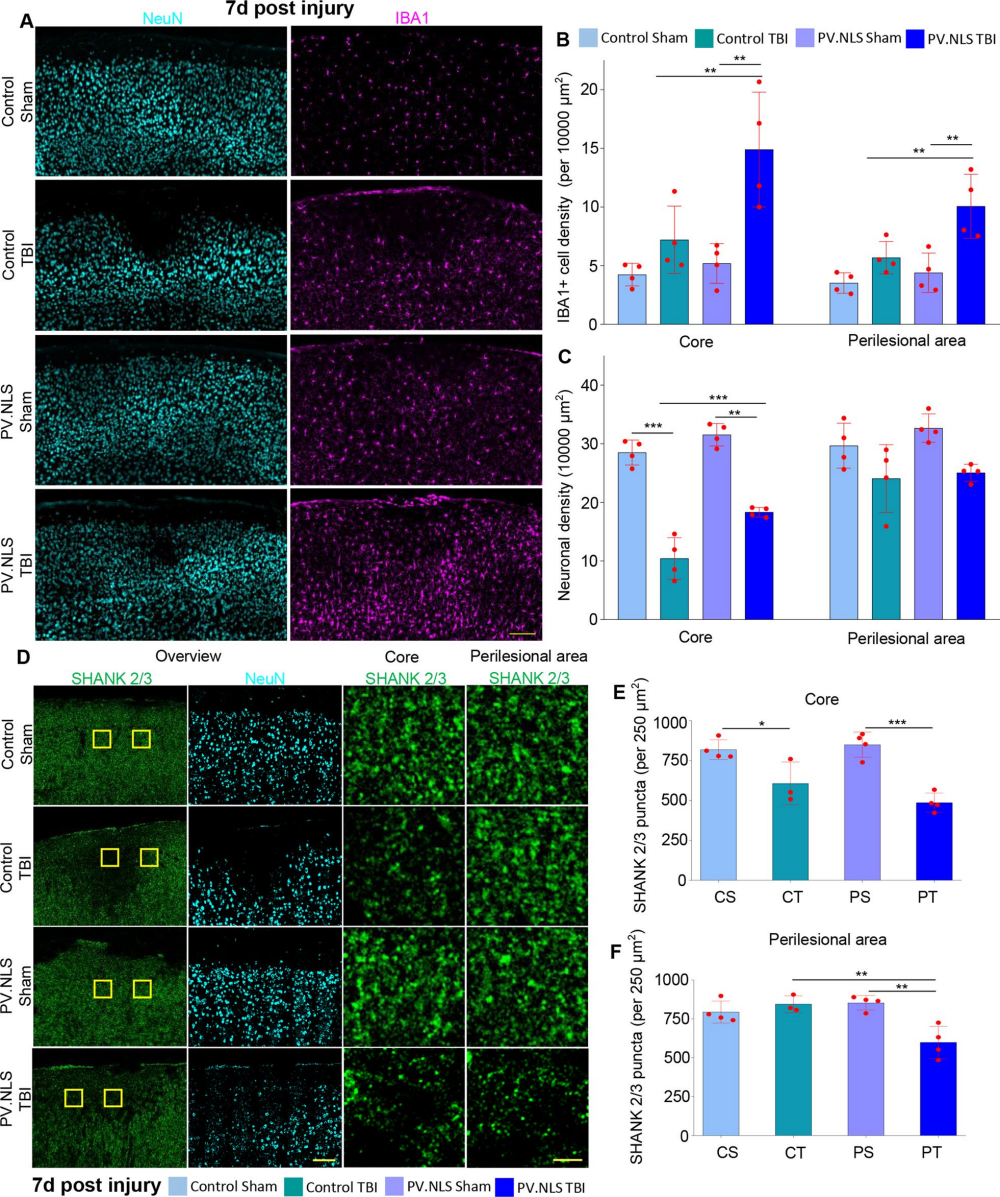
Buffering of neuronal nuclear calcium enhances subacute microgliosis and synapse loss 7 days post-trauma. **A**–**C** Buffering of nuclear calcium signaling in TBI significantly increased the density of IBA1 + cells compared to TBI alone in the core and perilesional areas (**B** CT vs PT; Core 7.20 ± 2.87 vs 14.90 ± 4.89; perilesional area 5.68 ± 1.37 vs 10.06 ± 2.72). Buffering of nuclear calcium signaling in TBI reduced the neuronal loss in the core (**C** CT vs PT; 10.42 ± 3.55 vs 18.32 ± 0.81), but not in the perilesional area (CT vs PT; 24.06 ± 5.78 vs 25.05 ± 1.44). Data are shown as mean ± SD. *N* = 4. Scale bar: 100 µm. **D**–**F** Significant decrease of excitatory synapses 7d post-TBI observed in animals injected with control AAV (CS vs CT; 819.12 ± 61.44 vs 607.12 ± 134.12) or nuclear calcium buffer (CT vs PT; 607.11 ± 134.52 vs 423.32 ± 60.11) in the core of the injury. Buffering of nuclear calcium signaling also resulted in a significant decrease in synaptic density in the perilesional area (CT vs PT; 845.2 ± 53.7 vs 599.2 ± 103.7). Data are shown as mean ± SD. *N* = 4.*p < 0.05; **p < 0.01; ***p < 0.001

At 7dpi, CT brains still displayed a slight accumulation of microglia, however their density remained substantially higher in PT sections both in the core and perilesional areas (Fig. 5A, B). Although there was little difference in neuronal loss between CT and PT both in the core and in the perilesional area (Fig. 5A, C), PT brains still displayed substantially reduced counts of synapses in the perilesional area (Fig. 5D-F), indicating the persistent larger area of synaptic loss which was already established at 24 h post-injury.

Taken together, these findings demonstrate that enhanced microgliosis resulting from NC buffering following TBI is not transient, but is maintained for up to 7 days and is associated with a larger area of synaptic loss, thus linking the robust cellular inflammatory response with synaptic damage.

### Blunting neuronal nuclear calcium worsens acute motor disturbances upon TBI

Next we explored the functional impact of the expanded, reactive microgliosis due to NC buffering after TBI. Since the somatosensory area of the brain provides strong excitatory drive to the primary and secondary whisker motor area [16, 43, 76], and based on evidence that silencing of the somatosensory area results in decreased whisking [63], we hypothesized that the disruption of synaptic networks in the somatosensory cortex might affect the whisking activity even in absence of a direct lesion of the motor area.

**Fig. 6:**
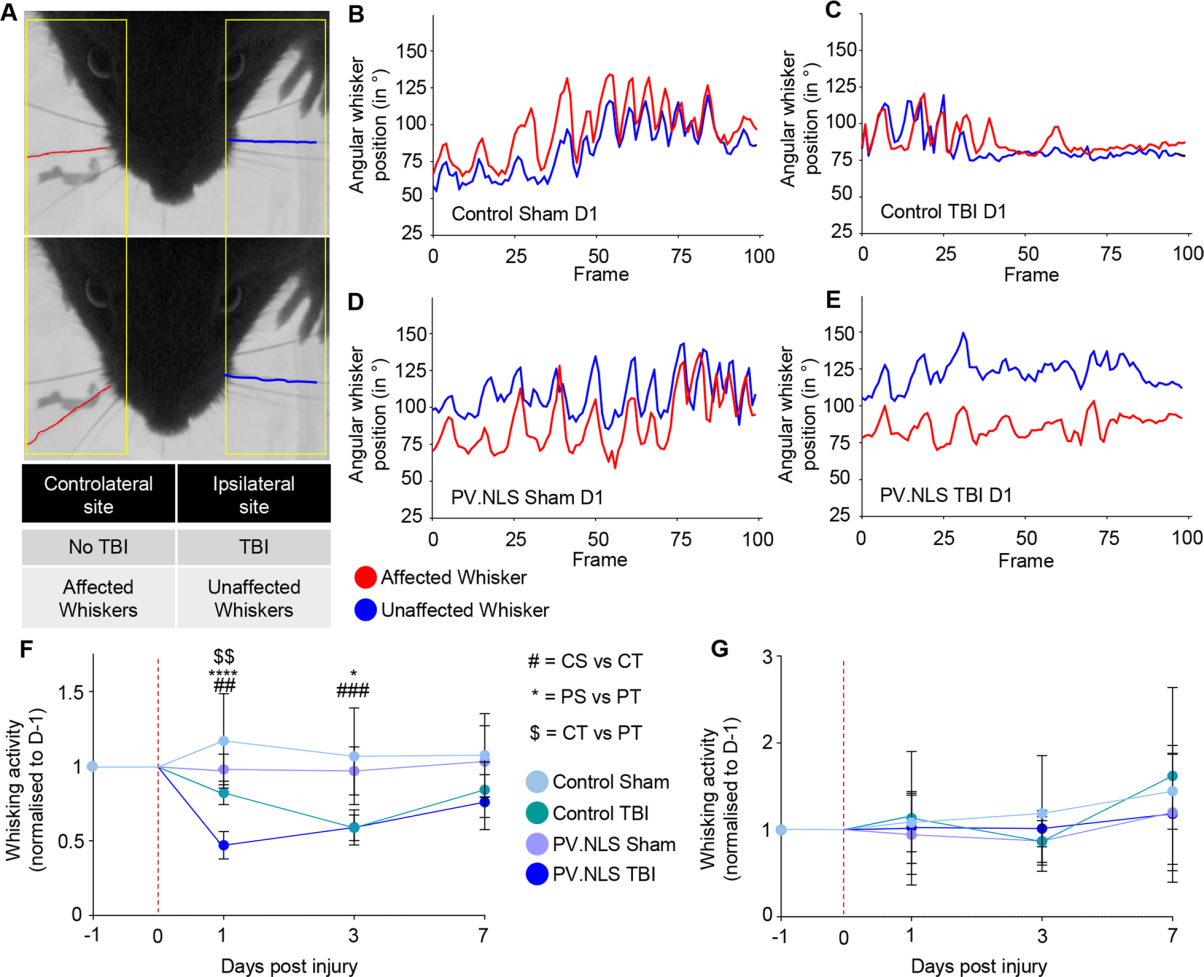
Blunting neuronal nuclear calcium worsens acute motor disturbances upon TBI. **A** Representative images of analyzed mouse whiskers, showing the affected (red) and unaffected (blue) whiskers. **B**–**E **Representative traces depicting the angular position of the affected whiskers (red) compared to the unaffected (blue) whiskers over time. Decreased activity is seen at D1 post-injury in PV.NLS TBI mice, but not in the other treatment groups. **F** Significant decrease of whisker activity after nuclear calcium buffering in TBI compared to TBI alone 1d post-TBI (CT vs PT; 0.82 ± 0.08 vs 0.47 ± 0.09). The difference is no longer detectable at later timepoints. **G** No significant changes to the whisking activity of the unaffected whisker was observed. *N* = 8 per group. *p < 0.05; **p < 0.01; ***p < 0.001; ****p < 0.0001

In fact, high-speed recordings of the spontaneous whisking activity of whiskers contralateral to the injury site assessed as number of whisking events (Fig. 6A) revealed that CT mice did show a decrease in spontaneous whisking already at 1 dpi which further deteriorated at 3dpi before showing a trend towards recovery at 7 dpi (Fig. 6B, C, F). While the whisking activity of PS mice were comparable to CS mice, PT mice displayed a significantly larger decline in whisking at 1 dpi compared to CT mice (Fig. 6C, E, F), indicating a more severe sensorimotor dysfunction in this group. However, the activity of PT mice converged with the CT counterparts by 3 dpi and with a similar recovery at 7 dpi. The kinetic parameters of single whisking events (Additional file 3: Fig. S3A–F) were comparable in the four groups, underscoring the sparing of the motor cortices; likewise, the activity of ipsilateral whiskers, controlled by the contralateral, uninjured side, was also comparable across the four groups (Fig. 6G). Of note, mice could not be tested at time points earlier than 1d because the stress of the TBI procedure made them characteristically uncooperative.

Thus, buffering of neuronal NC leads to a more severe acute disruption of the affected network.

### Targeted transcriptome analysis reveals new neuronal nuclear calcium-regulated mediators of neuro–glia crosstalk after TBI

**Fig. 7:**
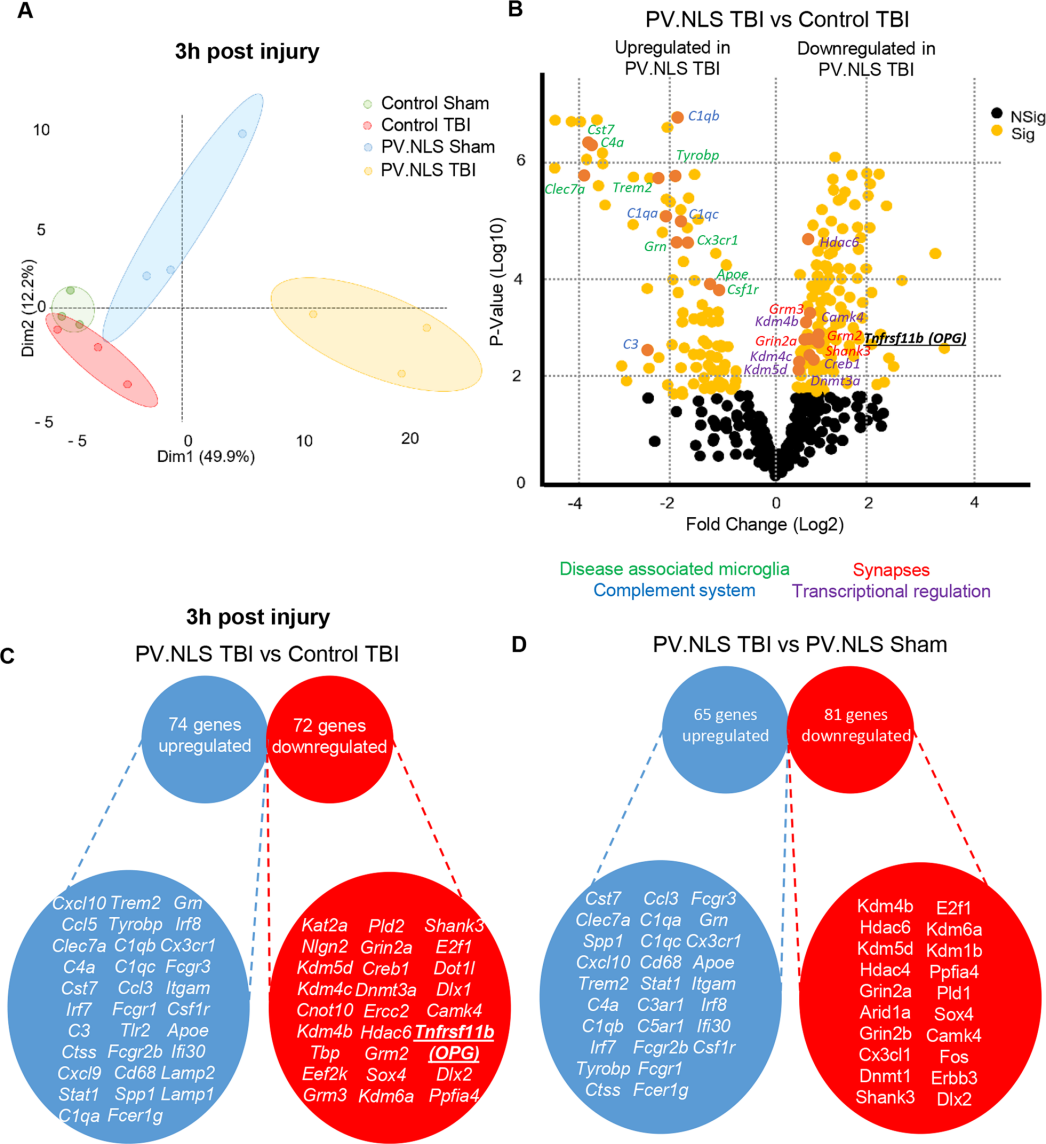
Targeted transcriptome analysis reveals new neuronal nuclear calcium-regulated mediators of neuro–glia crosstalk after TBI. **A** Principal component analysis (PCA) plot showing distinct clustering of each treatment. **B** Volcano plot of PV.NLS TBI vs control TBI shows upregulation of genes related to disease-associated microglia (green) or complement system (blue) and a down regulation of genes related to synaptic function (red) and transcriptional regulation (purple) 3 h after TBI. **C**, **D** A subset of the 74 genes upregulated and 72 genes downregulated in the PV.NLS TBI vs control (**C**) and of the 65 genes upregulated and 81 genes downregulated in the PV.NLS TBI vs PV.NLS sham (**D**) is depicted. A complete list of significantly differentially expressed genes can be found in Additional file 7: Table S2

In order to gain mechanistic insights into the processes triggering enhanced microgliosis and phagocytic phenotype occurring when neuronal NC is inhibited in TBI, we obtained a targeted nanostring transcriptome analysis of genes involved in neuroinflammatory cascades of the injected/injured area from CS, CT, PS and PT groups at 3 h post-injury. After pre-processing, quality control and normalization, the principal component analysis (PCA) distinctly defined the four groups (Fig. 7A), indicating significant differences in their transcriptome. Furthermore, the biological replications belonging to the four groups clustered together, segregated by treatment, in the unsupervised hierarchical clustering (not shown). The comparison of the transcriptome of CT vs PT samples (Fig. 7B, C) identified 74 upregulated and 72 downregulated genes (full list in Additional file 7: Table S2). Specifically, PT samples displayed a significant elevation in the expression of genes associated with phagocytic activity (*CD68, Lamp1, Lamp2, Itgam, Ctss, Tlr2, Ifi30*), antibody-mediated phagocytosis (*Fcgr1, Fcgr2b, Fcgr3, Fcer1g*) and in particular with DAM (*TREM2, Clec7a, Cst7, complement C4a, ApoE, Cx3cr1, Csf1r, Spp1, Tyrobp, Grn*). Furthermore, it showed a distinct elevation in the transcription of several other complement factors of the classical pathway (*C1qa, C1qb, C1qc, C3*), interferon-response genes *(Irf7, Irf8, STAT1)*, chemokines and other migration factors (*Ccl3, Ccl5, Cxcl9, Cxcl10*). Thus, PT samples displayed a transcriptome compatible with the histological evidence of increased microglial recruitment as early as 3 h after injury, with a DAM and phagocytic phenotype.

On the other hand, a number of genes associated with synaptic proteins (*Shank3, Grin2a, Grm2, Grm3*) or involved in transcriptional and epigenetic regulation (*Kdm4b and c, Kdm5d, Dnmt3a, Hdac6, CamKIV and Creb1*) were downregulated.

The comparison of the PT and PS transcriptomes revealed 65 upregulated and 81 downregulated genes (Fig. 7D). The list of upregulated and downregulated genes was remarkably similar to those that were revealed by the comparison of PT and CT transcriptomes, with a selective upregulation of complement-associated and DAM-associated genes (among others, *Clec7a, Cst7, C4a, Spp1, ApoE* as well as *C5ar1, C1qb, C1qa, C3ar1*), antibody-dependent phagocytosis and interferon-response genes. Among the downregulated genes, synaptic proteins and epigenetic regulators were also prominently represented. Taken together these findings suggest that the induction of DAM-like transcriptional signatures and downregulation of synaptic genes is not a consequence of NC blockade alone but a fingerprint of TBI occurring in the context of NC blockade.

### Neuronal expression of osteoprotegerin is upregulated by nuclear calcium signaling and neuronal activity in TBI

We interrogated the transcriptome dataset for soluble mediators, which may be potentially involved in neuron–microglia crosstalk, among the genes downregulated by PV.NLS upon TBI, under the hypothesis that the downregulation of one or more anti-inflammatory mediators may account for the upregulation of reactive microglia. We focused on *TNFRSF11b,* also known as osteoprotegerin (OPG), because (i) it is known to be released in the extracellular space and (ii) it reduces the activity of phagocytic cells in bone and connective tissue [26].

In fact, OPG is a soluble decoy receptor for RANKL, normally released in the extracellular space and readily detectable in biological fluids [26]; by antagonizing RANKL, OPG decreases osteoclast-dependent bone resorption. In the bone of OPG^−/−^ mice osteoclasts are overactive and OPG overexpression suppresses their phagocytic activity [13]. Interestingly, OPG also restricts microglial reactivity to bacterial infection [37].

**Fig. 8:**
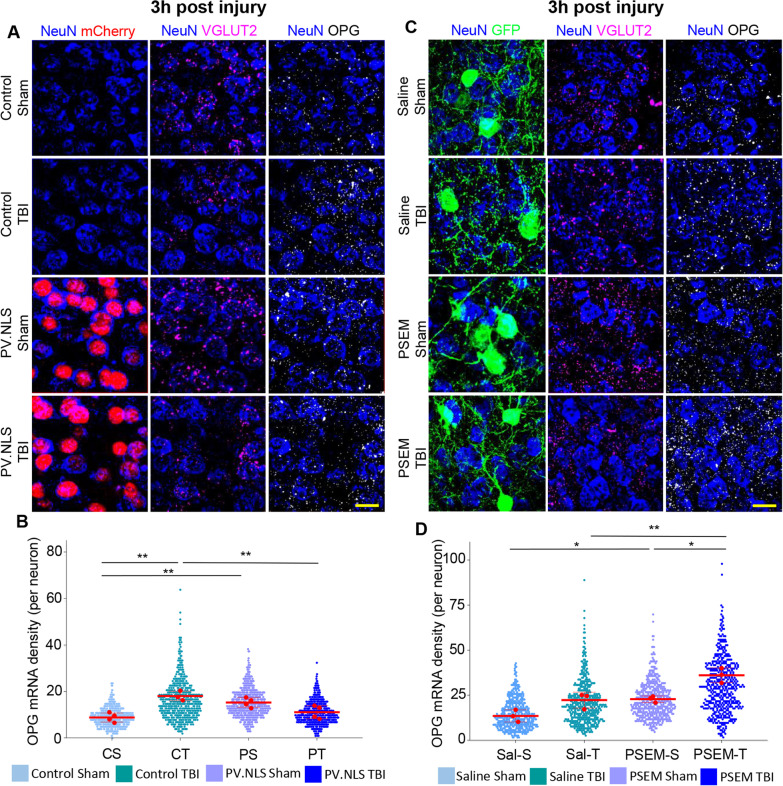
Neuronal expression of osteoprotegerin is upregulated by nuclear calcium signaling and neuronal activity in TBI. **A**, **B** Significant increase of OPG (TNFRSF11b) mRNA density 3 h post-TBI compared to sham (CS vs CT; 8.78 ± 1.95 vs 18.03 ± 2.08). Buffering of nuclear calcium suppresses the upregulation OPG mRNA upon TBI (CT vs PT; 18.03 ± 2.09 vs 11.06 ± 2.80). Small datapoints depict individual neurons, red datapoints depict average per animal (*N* = 4, used for statistics). **C**, **D** Chemogenetic inhibition of PV interneurons in TBI significantly increases OPG mRNA density compared to TBI alone (Sal-T vs PSEM-T; 22.24 ± 4.43 vs 31.77 ± 4.10). Note that all animals express the PSAM chemogenetic construct (green) but only the PSEM + groups receive the agonist. Small datapoints depict individual neurons, red datapoints depict average per animal (*N* = 3, used for statistics).Data are shown as mean ± SD *p < 0.05; **p < 0.01

In this context, we used single molecule in situ mRNA hybridization to confirm the modulation of OPG expression and pinpoint the cellular source of OPG. OPG mRNA colocalized with NeuN and VGLUT2 in all four treatment groups, indicating a neuronal source (Fig. 8A), further confirmed by the colocalization of 75% of OPG mRNA molecules with NeuN immunoreactivity (Additional file 4: Fig. S4B). Notably, the number of OPG mRNA molecules increased in neurons 3 h after TBI (Fig. 8A, C, D), but this upregulation was abolished in neurons expressing PV.NLS.mCherry (Fig. 8A, C). We further considered the expression level of OPG mRNA 24 h and 7 days after injury; at the former timepoint, a significant upregulation of OPG was detected in CT animals but not in PT animals, whereas at the latter only a non-significant trend was seen (Additional file 4: Fig. S4C–F). Thus, OPG upregulation is sustained between 3 and 24 h but goes back to baseline by 7 dpi.

We then wondered whether OPG expression could be enhanced by increasing neuronal firing through chemogenetic approaches. To achieve this goal, we injected an AAV encoding the inhibitory PSAM(Gly)-GFP construct into PV-Cre mice. PSAM(Gly)-GFP was successfully expressed in PV + interneurons (Fig. 8B). Upon PSEM administration, PSAM(Gly) decreased the excitability of PV interneurons, thereby reducing perisomatic inhibition of neighboring excitatory neurons and resulting in their increased firing [14]. Reduced PV firing (PSAM(Gly) + PSEM) resulted in the upregulation of OPG in sham mice and even more robustly in TBI mice, compared to control mice (PSAM(Gly) + vehicle; Fig. 8B, D)).

So it can be concluded that OPG is upregulated in neurons upon TBI through a process involving neuronal firing and NC signals.

### Re-expression of OPG in neurons with nuclear calcium buffering reduces microgliosis and prevents synaptic degradation after TBI

To establish a mechanistic link between neuronal OPG and TBI-induced microgliosis, we re-expressed OPG in neurons during NC blockade. The AAV expressing OPG under the hSyn promoter was sufficient to produce a massive upregulation of OPG expression irrespective of PV.NLS expression (Additional file 5: Fig. S5A, B). Mice were injected with a mix of AAV encoding either PV.NLS alone (PT) or PV.NLS and OPG (POT) 30 days before being subject to TBI. Two more groups were considered, both injected with AAV encoding an empty control vector, and subject to sham surgery (CS) or TBI (CT).

**Fig. 9:**
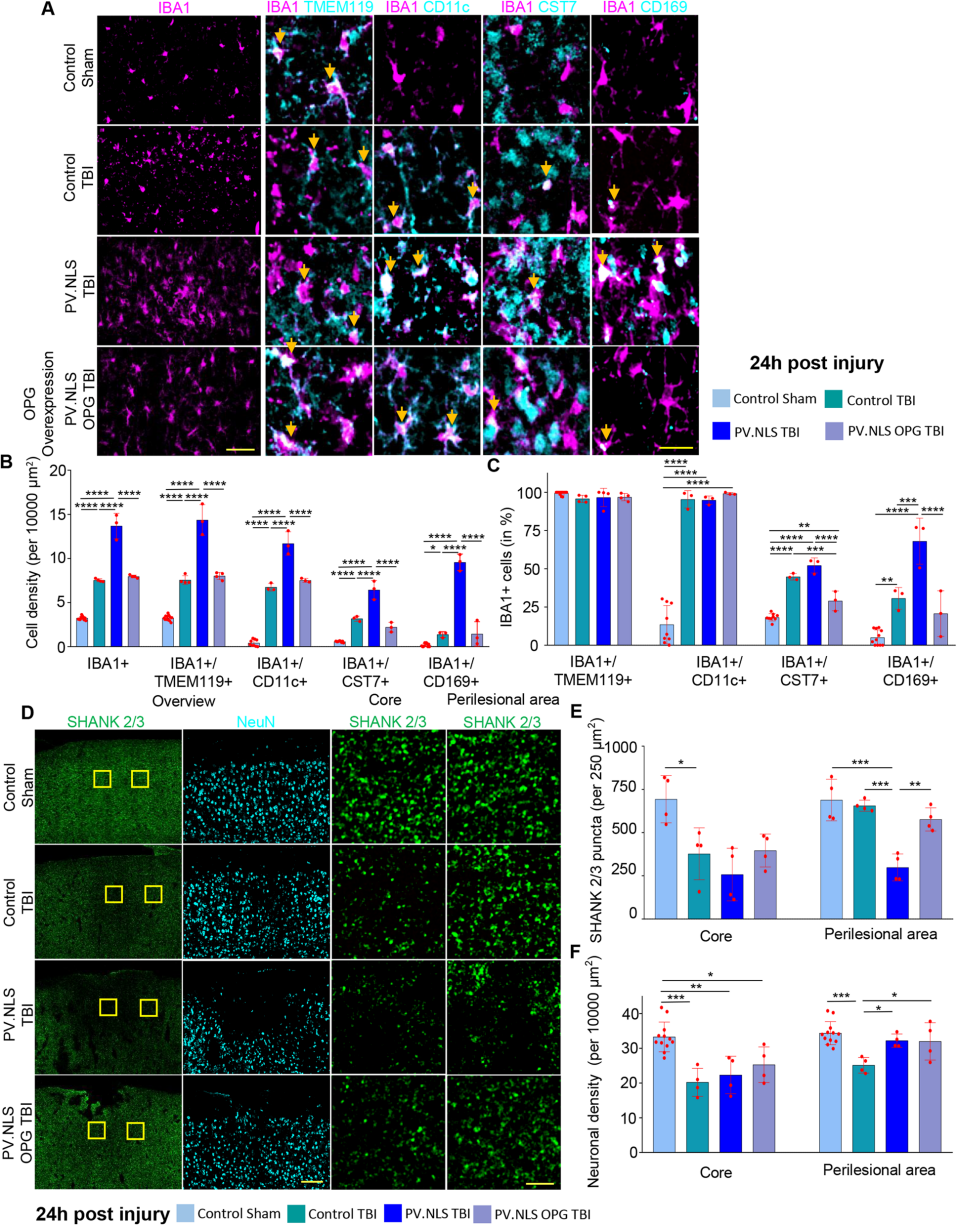
Re-expression of OPG in neurons with nuclear calcium buffering reduces microgliosis and prevents synaptic degradation after TBI. **A**–**C** Buffering of nuclear calcium signaling significantly increased the density of IBA1 + /TMEM119 + , IBA1 + /CD11c + , IBA1 + /CST7 + and IBA1 + /CD169 + cells 24 h after TBI compared to TBI alone (CT vs PT; for IBA1 + /TMEM119 + 7.56 ± 0.48 vs 14.37 ± 1.74; for IBA1 + /CD11c + 6.74 ± 0.42 vs 11.70 ± 1.33; for IBA1 + /CST7 + 3.17 ± 0.29 vs 6.44 ± 1.05; for IBA1 + /CD169 + 1.38 ± 0.329 vs 9.56 ± 0.934). Re-expression of OPG decreased IBA1 + /TMEM119 + , IBA1 + /CD11c + , IBA1 + /CST7 + ; IBA1 + /CD169 cells compared to PV.NLS TBI (PT vs POT; for IBA1 + /TMEM119 + cells 14.37 ± 1.74 vs 8.02 ± 0.40; for IBA1 + /CD11c + cells 11.7 ± 1.33 vs 7.52 ± 0.21; for IBA1 + /CST7 + cells 6.44 ± 1.05 vs 2.20 ± 0.54; for IBA1 + /CD169 + 9.56 ± 0.93 vs 1.44 ± 1.38). Likewise, re-expression of OPG normalized the fraction of IBA1 + /CST7 + and IBA1 + /CD169 + compared to PV.NLS TBI (PT vs POT; for CST7 + 52.14 ± 4.91% vs 28.98 ± 6.44%; for CD169 + 68.01 ± 15.06% vs 20.67 ± 14.95%). *N* = 3. Mean ± SD Scale bar: 40 µm (overview) and 20 µm (insert). **D**, **E** Re-expression of OPG together with buffering of nuclear calcium signaling did not alter core synaptic density compared to PV.NLS-TBI but significantly increased the synaptic density in the perilesional area compared to PV.NLS-TBI (PT vs POT; 298.3 ± 78.1 vs 576.3 ± 66.8). *N* = 4. **F** OPG re-expression together with nuclear calcium buffering did not alter core neuronal density compared to TBI alone but significantly increased neuronal density in the perilesional areas (CT vs POT 25.13 ± 2.26 vs 32.01 ± 5.38). Yellow inserts show analyzed core and perilesional areas. Scale bar: 100 µm (overview) and 5 µm (insert). Data are shown as mean ± SD. *N* = 4. *p < 0.05; **p < 0.01; ***p < 0.001

Re-expression of OPG substantially decreased the extent of microgliosis as determined by the density of IBA1 + and IBA1 + /TMEM119 + cells (Fig. 9A, B) and IBA1 + /CD11c + cells after TBI in POT mice compared to PT mice (Fig. 9A, B). Furthermore, OPG re-expression substantially decreased the proportion of cells expressing CST7 (Fig. 9A, C) as well as of CD169 + microglia (Fig. A, C), demonstrating that OPG re-expression is sufficient to normalize the recruitment and phenotype of microglia upon TBI.

Re-expression of OPG also normalized the synaptic density in the perilesional area, resulting in a smaller area of synaptic loss (Fig. 9D, E). It is worth noting that synaptic loss in the core was not affected by OPG expression, indicating that the damage caused by TBI was comparable in all injured groups. While neuronal density in the core of POT samples was similar in CT and PT samples, further confirming the reproducibility of TBI, and was comparable to PT in the perilesional area (Fig. 9D, F), indicating that while microgliosis and synaptic loss may be influenced by OPG, other aspects of the TBI pathophysiology may not be restored once the mechanical damage has occurred.

Thus, re-expression of OPG in neurons reverted the increased microgliosis and more extensive synaptic loss observed upon trauma when NC signaling was inhibited.

### OPG concentration increases in CSF samples from human TBI patients

Finally, we explored the translational value of OPG role in human TBI patients. We considered a series of CSF samples obtained from patients with severe TBI on the day of trauma (D0) and 1, 3 and 7 days later (D1, D3 and D7, respectively). Unfortunately, complete longitudinal CSF samples were not available and therefore distinct cohorts were considered. Samples from 6 non-TBI patients who required neurosurgery were included as baseline controls (clinical and demographic data are detailed in Table 1). The clinical cohort from which these samples were obtained has been the object of previous work [77]. The four TBI cohorts (D0, D1, D3, D7) did not differ in terms of age, Glasgow Coma Scale (GCS) at admission, Injury Severity Score (ISS) at admission and Glasgow Outcome Scale-Extended (GOSE) at 6 months (Table 1; the age of the individuals in the control group was significantly different from those of the cases.

**Table 1:** Demographic and clinical details for the CSF cohort

	Control group*	D0 group	D1 group	D3 group	D7 group	*p* value
*N* (M/F)	6 (2/4)	18 (12/6)	18 (15/3)	15 (11/4)	16 (12/4)	
Age (median, range)	71 (53–85)	35 (23–54)	36 (23–48)	31 (19–66)	28.5 (19–66)	0.43 (K-W)
Glasgow Coma Scale (median, range)	N/A	5.5 (3–10)	5.5 (3–10)	6.5 (3–10)	5.5 (3–11)	0.88 (K-W)
Injury Severity Score (median, range)	N/A	39 (17–50)	35 (18–59)	33.5 (17–58)	34.5 (17–50)	0.98 (K-W)
Glasgow Outcome Scale-Extended (median, range)	N/A	3 (1–8)	4 (1–8)	4 (1–8)	3.5 (1–8)	0.97 (K-W)

^*^ Non-TBI CSF samples were obtained from hydrocephalus patients (without previous TBI) undergoing implantation of ventriculoperitoneal shunts

**Fig. 10:**
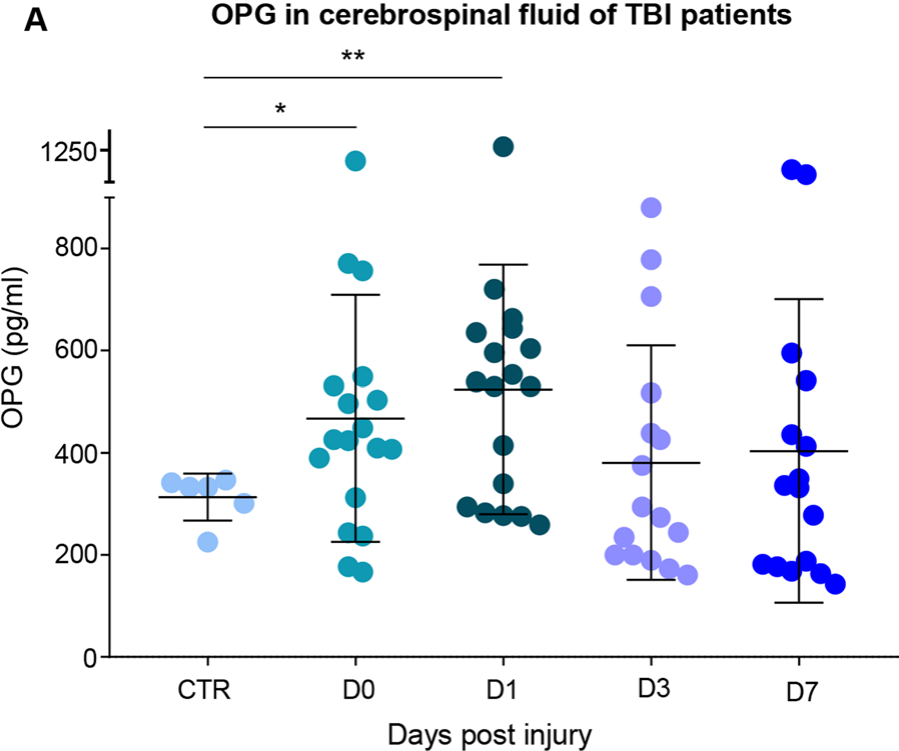
OPG concentration increases in CSF samples from human TBI patients. **A** Significant increase in osteoprotegerin (OPG) levels in cerebrospinal fluid (CSF) of TBI patients upon admission (CTR vs D0; 313.0 ± 46.1 pg/ml vs 467.5 ± 241.0 pg/ml) and 1d after trauma (CTR vs D1; 313.0 ± 46.1 pg/ml vs 524.1 ± 244.3 pg/ml). OPG levels decreased subsequently 3d (CTR vs D3; 313.4 ± 46.1 pg/ml vs 380.7 ± 229.6 pg/ml) and 7d (CTR vs D7; 313.4 pg/ml vs 403.6 ± 297.3 pg/ml) after trauma. Data are shown as mean ± SD. CTR *N* = 6; D0 *N* = 18; D1 *N* = 18; D3 *N* = 16; D7 *N* = 16. See Table 1 for clinico-demographic parameters. *p < 0.05; **p < 0.01

Compared to controls, levels of OPG at D0 were significantly elevated and displayed further increase at D1, which returned to baseline at D3 and D7 (Fig. 10).

This data supports a robust elevation of OPG in human CSF, with a peak in the early phase and followed by a rapid normalization, suggesting that OPG upregulation may have a pathophysiological role not only in rodents, but also in human TBI.

## Discussion

**Fig. 11:**
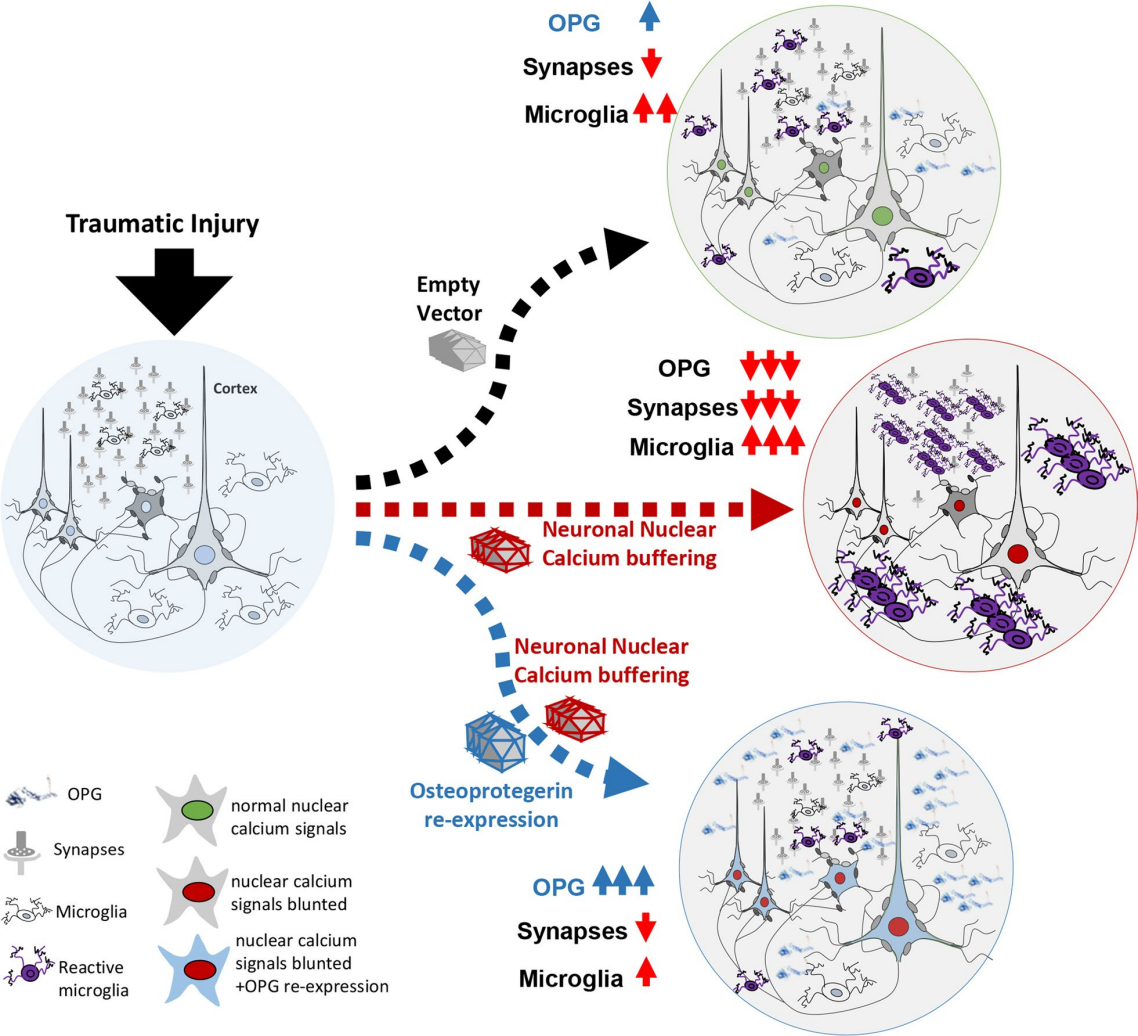
Neuronal nuclear calcium signaling controls neuroinflammation in TBI. In normal conditions (empty vector), neuronal activity induces a transcriptional program that limits the induction of reactive microglia upon TBI and the associated loss of synapses. When neuronal nuclear calcium is inhibited, however, synaptic loss and microglial recruitment are strongly increased upon TBI, but these effects are reversed by the re-expression of Osteoprotegerin (OPG)

In this study, we have demonstrated that neuronal NC signals are critical in keeping in check the activation of microglia following a mild TBI. Buffering of NC results in much greater recruitment of microglia displaying a typical disease-associated phenotype. Heightened microglia activation and accumulation was concomitant to a more profound loss of synapses, ultimately linked to a worse functional impairment. For the first time, we identify OPG as a neuronally originated, activity-dependent mediator of neuron–microglial crosstalk and an important player in restricting microglial reactivity and synapse elimination after TBI (summarized in Fig. 11). The possible translational value of our findings is supported by the elevation in OPG levels in CSF of TBI patients occurring within the first 24 h after trauma.

Our findings fit within the conceptual framework of neuron–microglia interaction [68]. It has previously been shown that decrease in neuronal activity obtained by sensory deprivation [41, 66] or by chemogenetics [67] results in the promotion of microglia motility and extension of processes probing the local environment, it is suggested [68] that enhanced microglial motility under neuronal hyperactivity may accelerate microglial response to local damage. In our experimental conditions, NC buffers (PV.NLS or CaMBP4 do not cause a decrease in neuronal activity per se, but are known to interfere with the transcriptional programs linked to neuronal activity [5, 12, 82, 83]. Thus, NC buffers generate a transcriptional landscape corresponding to neuronal hypoactivity,in agreement with previous findings, we observe an increased microglial reactivity to traumatic damage, both in terms of number and phenotype of the microglial cells.

Microglial response to hyperactivity has been linked to the increased purinergic signaling directly or indirectly related to neuronal release of ATP [20, 23] and to the secretion of chemokines from neurons (such as fractalkine, [22]); however, these mediators are not involved in the response to hypoactivity [41]. Microglial response to neuronal hypoactivity has been rather linked to a decrease in noradrenergic cAMP/PKA signaling occurring as consequence of lowered network activity [41, 46, 65]. Recently, BDNF has been also reported to be involved in neuron–microglia crosstalk, with decreased levels of BDNF correlated with enhanced microglial motility and synapse elimination [49], although it is not ascertained that this axis is involved in activity-dependent regulation of microglia.

In our experimental conditions, we did not observe a change in microglial CREB phosphorylation, possibly indicating that cAMP levels in microglia were not directly affected by the NC buffering in principal neurons. On the other hand, our transcriptome analysis and the re-expression experiments suggest that neuronal OPG may constitute an additional mediator (together with noradrenergic signaling and possibly BDNF and complement factors; [42]) linking neuronal activity and microglia.

OPG is a member of the TNF superfamily and it is more specifically part of the RANKL/RANK/OPG axis [26, 29]. RANK is the cognate receptor of the RANK ligand (RANKL), whereas OPG is a soluble decoy that binds to RANKL, effectively, downregulating RANK signaling. OPG was first identified for its function in bone metabolism: OPG prevents the RANK signaling and reduces osteoclast formation [69]. OPG expression in the brain has been previously reported [32, 37], but its role in microglial biology is currently controversial. OPG has been shown to be upregulated by ischemia in juvenile rats and mice [38, 61] and in a murine model of middle-cerebral artery occlusion, OPG deletion results in decreased infarct volume, reduced brain edema, downregulation of IL-6 and TNF-α, and diminished infiltration of macrophages [61]. In this stroke study, it was concluded that OPG signaling has an overall detrimental effect, leading to a pro-inflammatory phenotype in macrophages and consequent increased neuronal vulnerability [61]. On the other hand, OPG levels are reported to be reduced in multiple sclerosis (MS), a condition characterized by CNS inflammation [27]. So, it is speculated that OPG signaling may be protective in this autoimmune disease, by decreasing the activity of the RANK/RANKL [28]. Although the role of OPG in neuroinflammation may be highly context-dependent, it is worth noting that in non-cerebral tissues OPG has been shown to reduce inflammation. In fact, dendritic cells from OPG^−/−^ mice produce higher levels of inflammatory cytokines in vitro and in vivo upon LPS treatment [17] and are more effective in driving T cell responses [81]. Conversely, OPG administration ameliorates intestinal inflammation and mucosal dendritic cell infiltration [3], while OPG overexpression reduces dendritic cell activation in an asthma model [79]. Our data show that OPG downregulation is at least one of the factors leading to increased microglial reactivity when neuronal NC signaling is inhibited, since re-establishing OPG expression normalized microglial response to mild TBI and prevented the excessive loss of synapses. These findings are strongly consistent with the broader interpretation of OPG as a down regulator of immune activation [70].

Upon TBI, microglial cells in the context of neuronal NC blunting display a CD11c + /CST7 + and CD169 + phenotype. The expression of CD11c + and CST7 + in microglia has been originally associated with the DAM phenotype [36, 39] associated with degenerative diseases as well as traumatic brain injury [33]. Although often considered detrimental because of its association with synaptic loss and neuropathological features [18], CD11c + microglia has been also identified during development, aging and in association with white matter tracts [75]. Moreover, induction of CD11c + microglia by CSF1 has been associated with reduced demyelination in EAE [74] whereas blockade of CD11c + microglia is detrimental [52]. Thus, both protective and detrimental roles for CD11c + microglia have been proposed [9]. In our conditions, the upregulation of CD11c + , concomitant with the upregulation of the phagocytic microglia marker CD169 [11], is associated with an increased synaptic loss and a worsening of the acute neurological deficits, both the density of reactive microglia and the loss of synapses are reversed by the re-expression of OPG. Nevertheless, some beneficial roles of reactive microglia, such as in debris removal, cannot be discounted and are worth further investigation.

A few limitations of the study must be noted: first, we used only male mice for the experimental TBI; however, the well-established sex differences in TBI outcome and neuroinflammation [10] call for a further elucidation of neuronal activity mechanisms in female vs male mice. Second, the data from the human cohort correspond to a more severe population of cases (and a mix of male and female patients) than in the experimental conditions,however, acquisition of CSF samples from mild human TBI is ethically controversial, and although the human and mouse paradigms are not overlapping, the similarity of the OPG profiles suggests that OPG-related mechanisms may be unfolding in humans as well, and are therefore worth of closer inspection.

In conclusion, our data show that neurons displaying a normal NC signaling and sustained levels of neuronal function secrete OPG and other factors that restrain the local activation of microglia and the appearance of the DAM-like microglial phenotype. The overall physiological goal may therefore be to single out healthy neurons from the damaged ones and prevent the phagocytosis of the functioning neurons while enhancing it for those that have been damaged by the injury. This mechanism may have broader implications for inflammatory and neurodegenerative diseases characterized by disturbances in neuronal activity and NC signaling [59].

## Materials and methods

### Animals

B6;129P2-Pvalb^tm1(cre)Arbr^/J mouse line (Jax stock #008069) was a courtesy of Pico Caroni and Silvia Arber. All experiments were approved by the Tierforschungszentrum Ulm and by the Regierungspraesidium Tubingen with licence no. 1420. Only male mice were used in the present study. We elected to use the PV-Cre line for all experiments in order to have the same strain used for chemogenetic manipulations as well as for other manipulations with an uninterrupted quality control benchmark.

The PV-Cre animals were housed in either Type II long IVC cages or open cages in a group of 2–5 with ad libitum access to food and water, nest-building material and a day–night cycle of 12 h from 6:00 to 18:00.

### Virus production

AAV2/9 were prepared as previously described using the iodixanol density gradient protocol [16]. The helper plasmids pAd-DeltaF6 (James M. Wilson, Addgene 112867) and p5E18V2/9 (a kind gift from J. Kleinschmidt) were used for the production. The viral suspension was concentrated to a final volume of 200 µl and the titre (number of viral genomes/ml) was confirmed by qPCR. The following constructs were expressed: an empty vector hSyn.NLS.myc (negative control), hSyn-PV.NLS.mCherry, (previously reported by [55]), hSyn-CaMBP4.flag.mCherry (described by [62]), hSyn-TNFRSF11b-P2A-mCherry (Vigene biosciences); pAAV(9)-pCAG-A7-floxed-PSAM(L141F, Y115F)-GlyR-GFP-WPRE (inhibitory PSAM(GLY)-GFP construct; Vector Biolabs). The full list of constructs can be found in Additional file 8: Table S3.

### Intracerebral injection of AAV

Intracerebral injection of the AAV suspension was performed in mice aged p30-35 in correspondence of the primary somatosensory cortex, as previously described [14]. Briefly, mice were administered 0.05 mg/kg Buprenorphine and 1.0 mg/kg Meloxicam 30 min before being subject to sevoflurane anesthesia (5% in 95% O_2_ and then positioned in the stereotaxic frame. After the incision of the scalp, a burr hole was drilled in the parietal bone. The AAV suspension (1 µl, diluted 1:1 with 1% Fast-Green in DMEM was injected at the coordinates* x* =  + 2.0,* y* = -2.0, z = -0.2/0.6 using a pulled-glass capillary connected to a Picospritzer microfluidic apparatus. The injection procedure lasted approx. 10 min; the capillary was left in place for another 10 min. The scalp was then sutured with Prolene 7.0. Mice were administered with a daily dose of 1 mg/kg meloxicam and three daily doses of 0.05 mg/kg Buprenorphine for the next 3 days.

### Traumatic brain injury procedure

Traumatic brain injury (TBI) was performed as previously reported [14, 24, 50]. Animals were subjected to TBI at the age of P60–70. Briefly, animals were given sevoflurane anesthesia (4% Servofluran, 96% O_2_ and a dose of 0.05 mg/kg buprenorphine. The scalp was incised on the midline and the parietal bone exposed; the animals were then positioned in the TBI apparatus, while under anesthesia, so that the impact site corresponded to the injection site. The impact parameters were the following: 120 g weight, released from a height of 40 cm leading to a maximum skull displacement of 1.5 mm [14]. Immediately after the impact, mice received 100% O_2_ and the temporary apnea time was measured. The mice were kept under anesthesia (4% Servofluran, 96% O_2_) while the skin was sutured using Prolene 6.0 surgical thread. Mice received additional opiate (0.05 mg/kg buprenorphine) treatment for the next 24 h, access to soft food pellets and were checked for their well-being using a score sheet. Sham operated mice underwent the same treatment except for the impact injury.

The Neurological Severity Score (NSS, assessed as described below) was assessed after 3 h, 2 dpi, 5dpi and at 7 dpi as appropriate for the experimental design; throughout the experiments here reported, 240 NSS evaluations were performed on 120 individual animals at different timepoints; in only 5 instances a score of “1” was recorded; as such, no animal met the criteria for early killing (NSS ≥ 8). In Additional file 6: Table S1, we report the NSS scores for each individual animal at each available timepoint (depending on the timepoint of animal killing, we report either the 3 h score or the timecourse at 3 h, 2 days, 5 days and 7 days).

### Neurological severity score

The Neurological severity score (NSS) was measured at 3 h, 2 days, 5 days and 7 days post-TBI. NSS was scored as previously reported [24]. Briefly, mice were subjected to ten different tasks after each other with a break of 10–30 s between individual tasks to investigate neurological damage after TBI. In the first task mice had to escape an arena (30 cm diameter and 1 opening) within 3 min. Additionally, the mice were scored if they exhibit a mono- or hemiparesis in either of their limbs. The following set of tasks were observing the general behavior of mice, which consisted of checking their ability to walk straight, their search behavior and their startle reflex (triggered by sudden and loud hand clapping). Furthermore, the mice had to balance on a 7-mm-wide angular beam and a 5-mm-wide round beam for at least 10 s each. Finally, the mice had to succeed in a beam walk test with a beam length of 30 cm and a width of 3 cm, 2 cm and 1 cm. Every time a mouse failed to perform one of the tasks they were scored 1 point for each failure, allowing a total of 10 points for the Neurological severity score. The total NSS score for each animal can be found in Additional file 6: Table S1.

### Immunohistochemistry

Brain samples were processed as previously described [50]. Briefly, mice were killed by trans-cardial perfusion with 4% PFA in PBS, and brains were dissected and postfixed in 4% PFA overnight. Brains were then washed and transferred to 30% Sucrose for 2 days, after which the samples were embedded in OCT (Tissue Tek, Sakura, Germany). 40 micron sections were cut with a cryostat (Leica CM 1950 AG Protect cryostat). Sections spanning the injury site were selected and blocked (3% BSA, 0.3% Triton in 1 × PBS) for 2 h at room temperature, followed by incubation for 48 h at 4 °C with primary antibodies diluted in blocking buffer. Sections were washed 3 × 30 min with PBS, and incubated for 2 h at RT with secondary antibodies diluted in blocking buffer. The sections were washed 3 × 30 min with PBS and mounted using Prolong Gold Antifade Mounting Medium (Invitrogen, Germany). The full list of antibodies used can be found in Additional file 8: Table S3.

### Single molecule in situ hybridization

Single mRNA fluorescent in situ hybridization [72] was performed according to manufacturer instructions (ACDBio, RNAscope, fluorescent In Situ Hybridisation) for fixed frozen tissue sections, all reagents and probes were provided by ACDBio, Additional file 8: Table S3) with small modifications [50]. Briefly, cryosections were mounted in superfrost plus glass slides and frozen at − 80 °C for at least 24 h before the actual procedure. Sections were retrieved from the freezer and kept at RT before being washed in PBS for 5 min at RT. An antigen retrieval step was performed at 100 °C for 5 min, followed by washing twice in dH_2_0 and once in ethanol. The slides were then pretreated with reagent III for 30 min at 40 °C, and then washed twice in dH_2_0. Probe hybridization (OPG and VGLUT2) was performed for 4.5 h at 40 °C, followed by washing 2 × 2 min in washing buffer. First amplification was performed by incubating the slides in amplification-1 reagent for 30 min at 40 °C, followed by washing 2 × 2 min in washing buffer. Second amplification was performed by incubating the slides in amplification-2 reagent for 15 min at 40 °C, followed by washing 2 × 2 min in washing buffer. The last amplification was performed by incubating amplification-3 reagent for 30 min at 40 °C, followed by washing 2 × 2 min in washing buffer. The detection step was performed by incubating amplification-4 reagent for 45 min at 40 °C, followed by washing 2 × 10 min in washing buffer. Co-immunostaining was performed directly after the final detection, whereby the slides were blocked for 1 h in blocking buffer (3% BSA, 0.3% triton in 1 × PBS). Primary antibodies for GFP (Chicken; 1:500; Abcam Ab13970), NeuN (Guinea Pig; 1:250; SySy 266,004) and RFP (Camel; 1:250; Nanotech N0404-AT565-L) were diluted in blocking buffer and incubated overnight at 4 °C, followed by 3 × 30-min washing in PBS. Secondary antibodies for NeuN (Goat Anti-guinea pig IgG H&L Alexa Fluor 405; 1:250; abcam ab175678) and GFP (Alexa Fluor 488 AffiniPure Donkey Anti-Chicken IgY (IgG) (H + L); 1:250; Jackson 703-545-155) were diluted in blocking buffer and incubated for 2 h at RT, followed by 3 × 30 min washing in PBS. The sections were mounted using fluorogold ProLong antifade mounting medium (Invitrogen).

### Image acquisition and analysis

Confocal images were acquired in 1024 × 1024 pixel and 12-bit format, with a Leica DMi8 inverted microscope, equipped with an ACS APO 40 × oil objective. Parameters were set to obtain the signals from the stained antibody or mRNA and at the same time avoiding saturation. All fluorescent channels were acquired independently, to avoid cross-bleed. 3–4 sections spanning the core and perilesional area of the impact site were imaged of each mouse. Optical stacks of 30 micron were obtained and imported to ImageJ. For image analysis, stacks were collapsed in maximum intensity projection pictures and mean gray value or cell density per fixed region of interest (ROI) was measured.

For the quantification of the histological parameters, we considered a 200 µm × 200 µm ROI centered on the axis of the injury site (“core”) and two similar ROI located at 200 µm from the axis of the injury site (“perilesional area”; Additional file 2: Fig. S2A). The values obtained from the two perilesional area ROI were averaged for each section. In the samples not subject to trauma, we used the axis of the injection site as reference to establish the comparable ROIs for core and perilesional area.

CREB phosphorylation was visualized by acquiring a mosaic image of 2 × 1 optical stacks covering the center of the injection/injury site in layer II/III of the cerebral cortex. After importing the images to Image J a 200 µm × 200 µm area centered on the injury site was considered as ROI and mean gray value of pCREB was measured in every NeuN + or IBA1 + nucleus. To analyze the cell density mosaic images corresponding to 3 × 1 optical stacks spanning the core and perilesional area in the II/III cortical layer were acquired. After importing the images to ImageJ 200 µm × 200 µm ROIs were selected for analysis as indicated in Additional file 2: Fig. S2A and the cells within the ROIs were quantified. Furthermore only 1 optical slice at a depth of 15 microns was considered for determination of colocalization of multiple Antibodies. For OPG mRNA quantification a 2 × 2 mosaic image located in the core area of the injury in layer II/III was acquired. The images were imported to Image J and after maximum intensity projection, OPG mRNA density of each VGlut2 + Neuron was quantified.

Synaptic density was detected after producing a mosaic image corresponding to 6 × 6 single optical sections (acquired with a 63 × oil objective) with 1 μm thickness. Each cortical section was imaged at a fixed depth of 10 μm inside the section, the composite image was positioned so that an uninterrupted coverage of the impact site with perilesional area was acquired. Quantification of density of synapses was done with the IMARIS software (Bitplane AG, Zurich, CH), ROI were positioned at fixed distance into the cerebral cortex (layer II/III) at the core of the injury and at the fixed distance from the core (200 μm) at the perilesional area. The number of SHANK + synapses per unit area was counted using IMARIS as parameters; estimated XY diameter of 0.7 μm, quality of 28% and a region threshold of 0. The parameters were kept constant for each ROI.

### Nanostring targeted transcriptome and bioinformatic analysis

At 3 h post-TBI mice were euthanized, the brain quickly extracted. The cortex was dissected in ice cold PBS and snap frozen on dry ice. Cortical tissue was stored at − 80 °C until further use. RNA was extracted using the ISOLATE II RNA/DNA/Protein kit (Bioline, Germany) following the manufacturer's instructions. Reagents and buffers were all provided by the company. Briefly, cortical samples were homogenized in 300 μl lysis buffer, loaded on a DNA column and centrifuged at 14,000*g* for 1 min. The flow-through was collected and adjusted with ethanol before loading it on the RNA column and centrifuged at 3500*g* for 1 min. The column was washed 3 × by adding 400 μl of washing buffer and centrifuging at 14,000*g* for 1 min, followed by a dry spin at 14,000*g* for 2 min. The RNA was eluted by adding 50 μl of elution buffer and centrifuging at 200*g* for 2 min followed by a final dry spin at 14,000*g* for 1 min. The isolated RNA was collected and stored at − 80 °C until further use. The RNA concentration and quality were determined with the Nanodrop 2000 and samples were sent to Proteros (Germany) for gene expression profiling using NanoString technology.

The raw data files were loaded in R software and the dataset for each sample was preliminary subjected to quality control assessment (QCA). Normalized data were then subjected to principal component analysis (PCA) to display group-based clustering. Confidence ellipses (assuming multivariate normal distribution) with the first two principal components were plotted to validate further analysis. Modified linear modeling-based analysis was then applied to the data to identify RTK showing a significant increase or decrease in expression at different conditions. All significantly differentially regulated genes between the PV.NLS TBI (PT) and Control TBI (CT) or PV.NLS Sham (PS) are listed with name, log FC and their adjusted p-value in Additional file 7: Table S2.

### Whisking analysis

Whisking analyses were performed as previously reported [71]. Prior to the TBI the mice were trained on a daily basis for 7 days to acclimatize with the recording set-up, process and experimenter. Before the first training session, whiskers on both sides were trimmed, leaving only the γ whiskers intact. Each session started with a short acclimatization period followed by 4 recordings 50 s each, interleaved with 1–2 min breaks to reduce the stress level of the mice. For each recording, mice were manually held down on a platform, to reduce head movement, while allowing free whisker movement; the camera was positioned 20 cm above the mouse head. The head of the mouse was back-illuminated so that whiskers would appear black against the light background of the platform. The whisking was recorded using a high-speed camera (Baler acA1300-60gc) recording 100 frames per second (100 Hz) with a resolution of 900 × 450 pixels. Spontaneous whisking activity was assessed by counting the number of whisking events, focusing on the 3rd whisker of the γ row: the video recordings were logged in imageJ, slowed down by a factor of 25 and each whisking event was manually annotated. For a deeper kinetic analysis representative 1 s fragments were extracted from each recording; the target whisker and the reference point were manually traced and angular position to the skin surface, whisking speed and whisking amplitude of the whisking event were measured with the Templo and Vicon Motus 2D software (CONTEMPLAS, Germany) and logged as excel files.

### Clinical cohort and ELISA assay for OPG measurements

The collection of CSF samples from human TBI patients was authorized by The Alfred Hospital Ethical Committee (Melbourne) no. 194-05 to Cristina Morganti-Kossmann and by the Ulm University Ethical Committee no. 12/19-22050. Clinical and demographic characteristics of the patients are reported in Table 1. Patients for this cohort were recruited at the Alfred Hospital, Melbourne; informed consent was obtained from the next of the kin. Inclusion criteria were: severe TBI with a post-resuscitation GCS ≤ 8 (unless initial GCS > 9 was followed by deterioration requiring intubation) and, upon CT imaging, the need for an extraventricular drain (EVD) for ICP monitoring and therapeutic drainage of CSF. CSF was collected over 24 h and kept at 4 °C; samples were obtained on admission (day 0) and daily up to day 7 after injury. Within an hour from collection, samples were centrifuged at 2000 g for 15 min at 4 °C and stored at -80 °C until analysis. Exclusion criteria comprised pregnancy, neurodegenerative diseases, HIV and other chronic infection/inflammatory diseases, or history of TBI. Out of the 42 TBI patients constituting the original cohort [77], we selected a total of 67 samples corresponding to D0, D1, D3 and D7 after injury.

OPG in the CSF was measured using the Human TNFRSF11B (OPG) Elisa kit (Thermo Fisher Scientific, Germany). The assay was performed according to the manufacturer’s instructions. 100 µL of diluted sample (1:1 in the sample diluent) was used. Absorbance at 450 nm was measured in ELx800 Microplate Reader (BioTek) and concentrations were calculated according to the standard curve.

### Statistics

Statistical analysis was performed using GraphPad Prism version 8 software (GraphPad software, USA). The Shapiro–Wilk test was performed to test groups for normality. Grouped analysis was performed with the mean of each animal and using two-way ANOVA (analysis of variance) with Tukey’s multiple correction. Depending on normality, grouped analysis for the ELISA data was performed using ANOVA with Tukey’s multiple correction or Kruskal–Wallis test with Dunn’s multiple correction. Data are depicted as histograms with mean ± SD or scatterplot with mean ± SD. Statistical significance was set at *p* < 0.05. The statistical information for each figure is recorded in Additional file 9: Table S4.

## Supplementary Information

**Additional file 1: Figure S1.** Glial CREB phosphorylation post injury is limited to IBA1+ cells. A)-B) AAVs are able to infect 89.98 ± 14.47% of cortical neurons. Data are shown as mean ± SD (Red dots represent individual animals). N=8. Scale bar: 50µm. C)-D) Non-neuronal pCREB signal is mainly found in IBA1+ cells (IBA1+ cells: 97.76 ± 0.83% vs. GFAP+ cells: 1.24 ± 0.83%). Data are shown as mean ± SD. N=4. Scale Bar: 50µm.E-F) Significant increase of IBA1+ cell density in PV.NLS TBI treated mice compared to all other treatment groups (CS = 3.376 ± 0.3593; CT = 3.715 ± 0.7015; PS = 4.056 ± 0.9741; PT = 6.952 ± 1.57). Data are shown as mean ± SD. N=4. **: p < 0.01. Scale Bar: 25µm.

**Additional file 2: Figure S2.** Blunting neuronal nuclear calcium signaling did not alter IBA1+/TMEM119+ cell density and percentage in the perilesional area post-TBI. A) Depiction of the regions of interest: the core area of the injury and the perilesional areas 200µm away from the injury axis. Scale bar 200µm. B) No significant difference in IBA1+ and IBA1+/CD169+ cell density in the perilesional area 24h post injury (IBA1+: CS= 4.10 ± 0.47, CT = 6.73 ± 1.62 and PT = 8.92 ± 1.84; IBA1+/CD169+ CS = 0.18 ± 0.25, CT = 1.12 ± 1.48 and PT = 1.62 ± 1.44). Significant increase in IBA1+/CD11c+ cell density after TBI but unaltered by nuclear calcium buffering (CS vs CT 0.5 ± 0.39 vs 5.5 ± 1.77; CT vs PT 5.5 ± 1.77 vs 5.5 ± 0.97). IBA1+/CST7+ cell density is significantly increased by nuclear calcium buffering (CT vs PT 1.25 ± 0.3361 vs 3.175 ± 0.4787) Data are shown as mean ± SD. N=4. *p<0.05, **p<0.01; ***: p < 0.001. C) No significant differences in fractions of TMEM119+ and CD169+ cells 24h post injury (TMEM119+: CT vs. PT 95.91 ± 2.5% vs PT = 97.69 ± 0.87%; CD169+: CT vs PT 29.29 ± 41.73% vs 22.68 ± 20.96). Significant increase in CD11+ expression 24h after injury but unaltered by PV.NLS (CS vs CT 12.17 ± 10.2% vs 80.3 ± 8.32%; CT vs PT 80.3 ± 8.32% vs 64.44 ± 20.57%). Significant increase of CST7+ expression 24h after injury after nuclear calcium buffering (CT vs PT 28.19 ± 5.235% vs 50.32 ± 15.8%). Data shown as mean ± SD. N=4. **p<0.01, ***p<0.001. D)-F) Significant decrease of inhibitory (Gephryn) synaptic density 24h post-TBI in the core (CS vs CT; 870.5 ± 99.78 vs 574 ± 70.83) and in the perilesional area (CS vs CT; 1021 ± 161.5 vs 701.6 ± 31.64). Buffering of nuclear calcium signaling blunted the synaptic loss in the core (CT vs PT; 574 ± 70.83 vs 681.8 ± 74.02) but did not alter the synaptic density in the perilesional area (CT vs PT; 701.6 ± 31.64 vs 755.5 ± 85.07). Data are shown as mean ± SD. N = 4. **: p < 0.01; ****: p < 0.0001. Scale bar 100µm (overview) and 5µm (insert).

**Additional file 3: Figure S3.** Blunting neuronal nuclear calcium signaling did not alter amplitude and velocity of whisker movement post-TBI. A)-C) The amplitudes of the affected (CS = 0.88 ± 0.22; CT = 1.1 ± 0.19; PS = 0.93 ± 0.42; PT = 1.269 ± 0.56) or unaffected (CS = 1.07 ± 0.39; CT = 1.05 ± 0.35; PS = 1.14 ± 0.45; PT = 1.22 ± 0.45) whisker were not significantly changed 24h after injury. The Amplitude ratio between affected and unaffected whiskers (CS = 0.77 ± 0.27; CT = 1.24 ± 0.42; PS = 0.91 ± 0.45; PT = 1.10 ± 0.55) also remained unaltered. D)-F) Velocities of the affected (CS = 1.1 ± 0.38; CT = 1.02 ± 0.29; PS = 0.95 ± 0.43; PT = 1.43 ± 0.78) and unaffected (CS = 0.95 ± 0.66; CT = 0.82 ± 0.26; PS = 1.33 ± 0.75; PT = 1.2 ± 0.62) whisker or their ratio (CS = 1.74 ± 1.26; CT = 1.27 ± 0.72; PS = 0.89 ± 0.54; PT = 0.75 ± 0.33) are not significantly changed 24h after injury. Data shown as mean ± SD. N=7 in A-F.

**Additional file 4: Figure S4.** Neuronal expression of osteoprotegerin is upregulated by nuclear calcium signaling and neuronal activity in TBI. A) Negative Control, baseline OPG (TNFRSF11B) signal and a positive Control of the in situ hybridization 24h post injury. B) OPG (TNFRSF11B) in situ signal is mainly found in neuronal sources (neuronal vs non-neuronal 75.38 ± 1.83% vs 24.62% ± 1.83%). N=5. C)-D) Significant increase of OPG (TNFRSF11b) mRNA density 24h post-TBI compared to sham (CS vs CT; 8.53 ± 2.45 vs 28.74 ± 12.03). Buffering of nuclear calcium in TBI significantly decreased OPG mRNA density (CT vs PT; 28.74 ± 12.03 vs 12.14 ± 1.58). Small datapoints depict individual neurons, red datapoints depict average per animal mean ± SD. N=4 (used for statistics). Scale bar: 20µm. E)-F) Buffering of nuclear calcium in TBI significantly decreased OPG mRNA density (CT vs PT; 13.46 ± 5.41 vs 6.03 ± 1.75).mall datapoints depict individual neurons, red datapoints depict average per animal mean ± SD. N=4 (used for statistics). *: p < 0.05; **: p < 0.01. Scale bar: 20µm.

**Additional file 5: Figure S5.** Re-expression of osteoprotegerin together with nuclear calcium buffering massively induces neuronal OPG mRNA intensity. A)-B) Significant increase in neuronal OPG (TNFRSF11b) mRNA intensity (CS vs CT; 181.8 ± 24.34 vs 224.3 ± 35.87). Buffering of nuclear calcium signaling decreased neuronal OPG mRNA intensity (CT vs PT; 224.3 ± 35.87 vs 184.1 ± 24.11). Re-expression of OPG together with nuclear calcium buffering increased the neuronal OPG mRNA intensity massively (PT vs POT; 184.1 ± 24.11 vs 1386 ± 435). Each dot represents one neuron. N=4. Scale bar overview: 20µm.

**Additional file 6: Table S1.** Neurological Severity Score. All results of the Neurological Severity Score (NSS) are listed for each experimental cohort as total scores per mice in each treatment. Mice killed at either 3h or 24h had their NSS scored at 3h. Mice killed at 7d had their NSS scored at 3h, 2d, 5d and 7d.

**Additional file 7: Table S2.** Targeted transcriptome analysis reveals new neuronal nuclear calcium-regulated mediators of neuro–glia crosstalk upon TBI. All significantly differentially regulated genes between the PV.NLS TBI (PT) and Control TBI (CT) /PV.NLS Sham (PS) are listed with name, log FC and their adjusted p-value.

**Additional file 8: Table S3.** Antibodies and Probes. Full information on antibodies and probes is provided.

**Additional file 9: Table S4.** Statistical Summary. Full information on statistical analysis for each experiment has been listed.

## Data Availability

Raw data are available on request; the Nanostring targeted transcriptomics dataset is provided as supplementary excel file.
