# Supplementary figures and images for: Neuronal nuclear calcium signaling suppression of microglial reactivity is mediated by osteoprotegerin after traumatic brain injury
Source: J Neuroinflammation. 2022 Nov 19;19:279. doi: 10.1186/s12974-022-02634-4 (PMC9675197; doi:10.1186/s12974-022-02634-4)

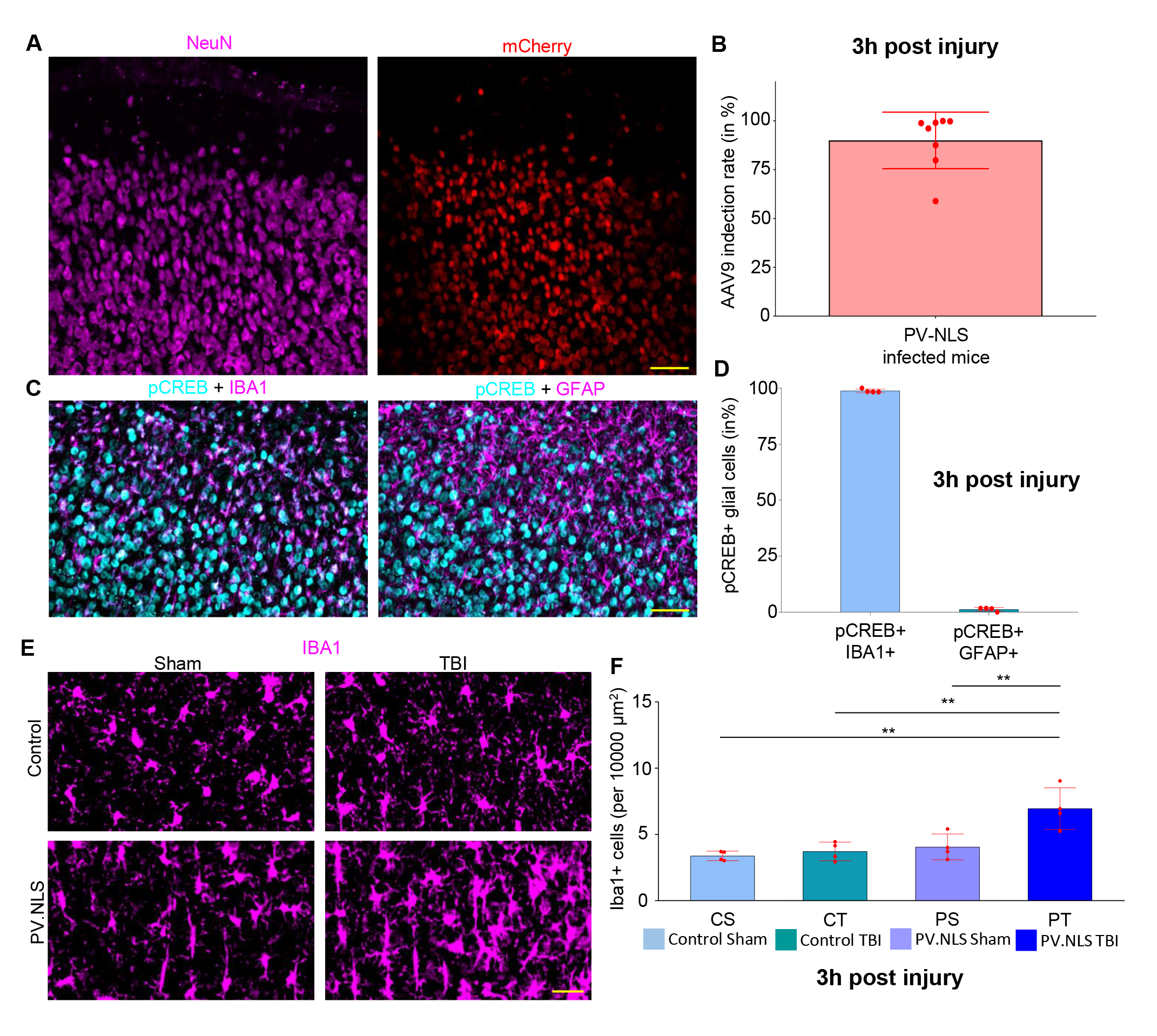

Supplement: Supplementary file 1 — Additional file 1: Figure S1. Glial CREB phosphorylation post injury is limited to IBA1+ cells. A)-B) AAVs are able to infect 89.98 ± 14.47% of cortical neurons. Data are shown as mean ± SD (Red dots represent individual animals). N=8. Scale bar: 50µm. C)-D) Non-neuronal pCREB signal is mainly found in IBA1+ cells (IBA1+ cells: 97.76 ± 0.83% vs. GFAP+ cells: 1.24 ± 0.83%). Data are shown as mean ± SD. N=4. Scale Bar: 50µm.E-F) Significant increase of IBA1+ cell density in PV.NLS TBI treated mice compared to all other treatment groups (CS = 3.376 ± 0.3593; CT = 3.715 ± 0.7015; PS = 4.056 ± 0.9741; PT = 6.952 ± 1.57). Data are shown as mean ± SD. N=4. **: p < 0.01. Scale Bar: 25µm. [file 12974_2022_2634_MOESM1_ESM.tif]

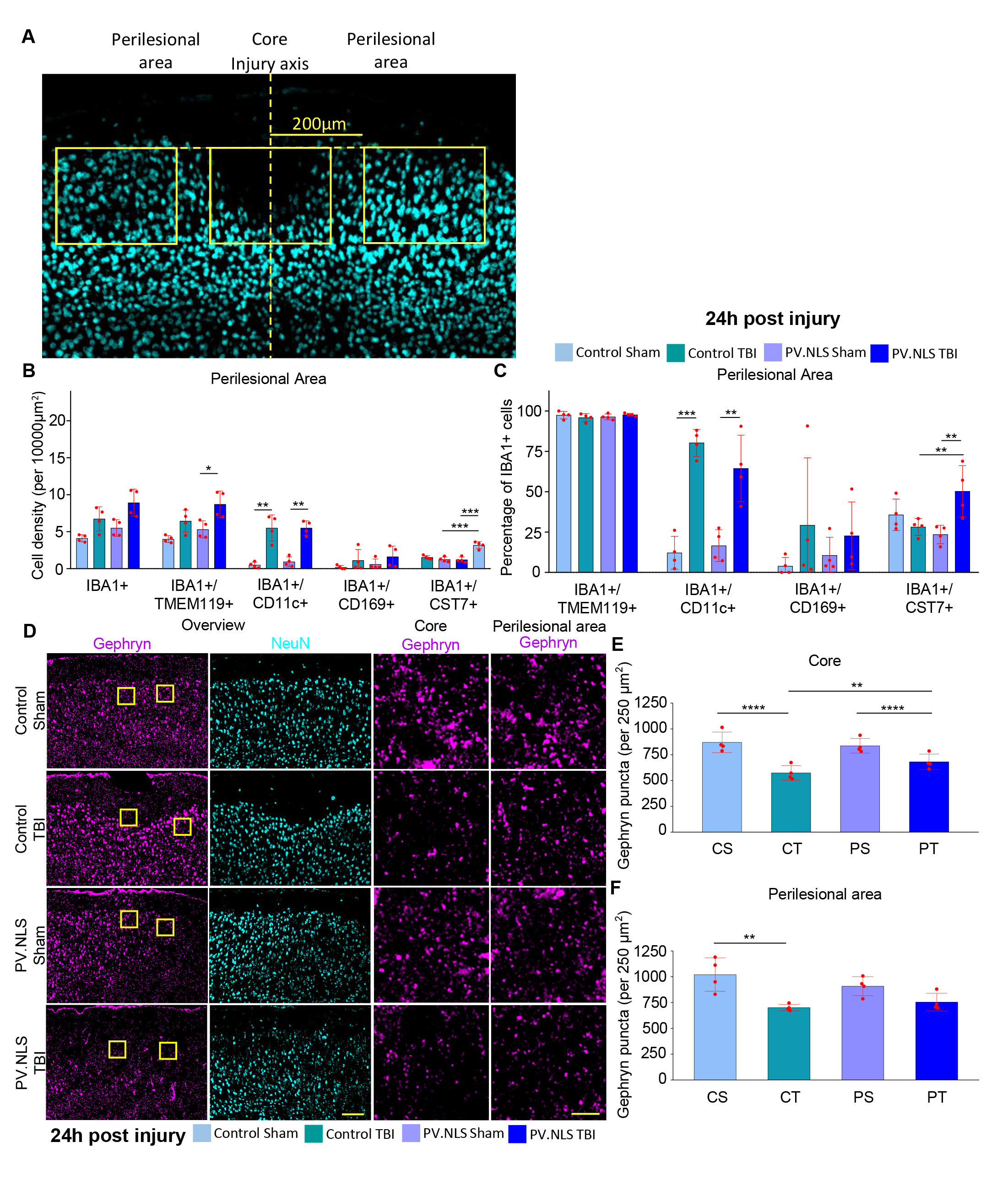

Supplement: Supplementary file 2 — Additional file 2: Figure S2. Blunting neuronal nuclear calcium signaling did not alter IBA1+/TMEM119+ cell density and percentage in the perilesional area post-TBI. A) Depiction of the regions of interest: the core area of the injury and the perilesional areas 200µm away from the injury axis. Scale bar 200µm. B) No significant difference in IBA1+ and IBA1+/CD169+ cell density in the perilesional area 24h post injury (IBA1+: CS= 4.10 ± 0.47, CT = 6.73 ± 1.62 and PT = 8.92 ± 1.84; IBA1+/CD169+ CS = 0.18 ± 0.25, CT = 1.12 ± 1.48 and PT = 1.62 ± 1.44). Significant increase in IBA1+/CD11c+ cell density after TBI but unaltered by nuclear calcium buffering (CS vs CT 0.5 ± 0.39 vs 5.5 ± 1.77; CT vs PT 5.5 ± 1.77 vs 5.5 ± 0.97). IBA1+/CST7+ cell density is significantly increased by nuclear calcium buffering (CT vs PT 1.25 ± 0.3361 vs 3.175 ± 0.4787) Data are shown as mean ± SD. N=4. *p<0.05, **p<0.01; ***: p < 0.001. C) No significant differences in fractions of TMEM119+ and CD169+ cells 24h post injury (TMEM119+: CT vs. PT 95.91 ± 2.5% vs PT = 97.69 ± 0.87%; CD169+: CT vs PT 29.29 ± 41.73% vs 22.68 ± 20.96). Significant increase in CD11+ expression 24h after injury but unaltered by PV.NLS (CS vs CT 12.17 ± 10.2% vs 80.3 ± 8.32%; CT vs PT 80.3 ± 8.32% vs 64.44 ± 20.57%). Significant increase of CST7+ expression 24h after injury after nuclear calcium buffering (CT vs PT 28.19 ± 5.235% vs 50.32 ± 15.8%). Data shown as mean ± SD. N=4. **p<0.01, ***p<0.001. D)-F) Significant decrease of inhibitory (Gephryn) synaptic density 24h post-TBI in the core (CS vs CT; 870.5 ± 99.78 vs 574 ± 70.83) and in the perilesional area (CS vs CT; 1021 ± 161.5 vs 701.6 ± 31.64). Buffering of nuclear calcium signaling blunted the synaptic loss in the core (CT vs PT; 574 ± 70.83 vs 681.8 ± 74.02) but did not alter the synaptic density in the perilesional area (CT vs PT; 701.6 ± 31.64 vs 755.5 ± 85.07). Data are shown as mean ± SD. N = 4. **: p < 0.01; ****: p < 0.0001. Scale bar 100µm (overview) an [file 12974_2022_2634_MOESM2_ESM.tif]

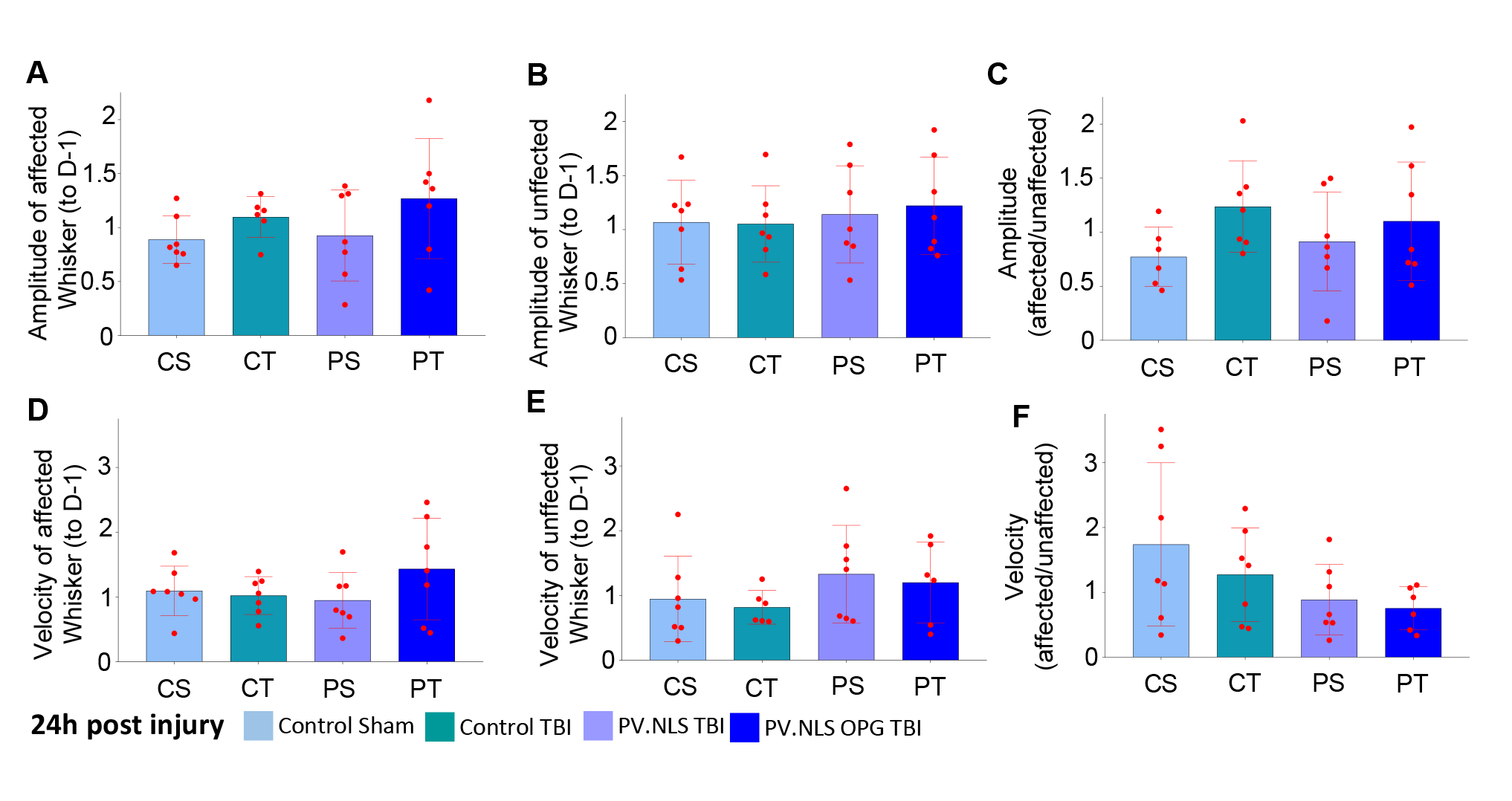

Supplement: Supplementary file 3 — Additional file 3: Figure S3. Blunting neuronal nuclear calcium signaling did not alter amplitude and velocity of whisker movement post-TBI. A)-C) The amplitudes of the affected (CS = 0.88 ± 0.22; CT = 1.1 ± 0.19; PS = 0.93 ± 0.42; PT = 1.269 ± 0.56) or unaffected (CS = 1.07 ± 0.39; CT = 1.05 ± 0.35; PS = 1.14 ± 0.45; PT = 1.22 ± 0.45) whisker were not significantly changed 24h after injury. The Amplitude ratio between affected and unaffected whiskers (CS = 0.77 ± 0.27; CT = 1.24 ± 0.42; PS = 0.91 ± 0.45; PT = 1.10 ± 0.55) also remained unaltered. D)-F) Velocities of the affected (CS = 1.1 ± 0.38; CT = 1.02 ± 0.29; PS = 0.95 ± 0.43; PT = 1.43 ± 0.78) and unaffected (CS = 0.95 ± 0.66; CT = 0.82 ± 0.26; PS = 1.33 ± 0.75; PT = 1.2 ± 0.62) whisker or their ratio (CS = 1.74 ± 1.26; CT = 1.27 ± 0.72; PS = 0.89 ± 0.54; PT = 0.75 ± 0.33) are not significantly changed 24h after injury. Data shown as mean ± SD. N=7 in A-F. [file 12974_2022_2634_MOESM3_ESM.tif]

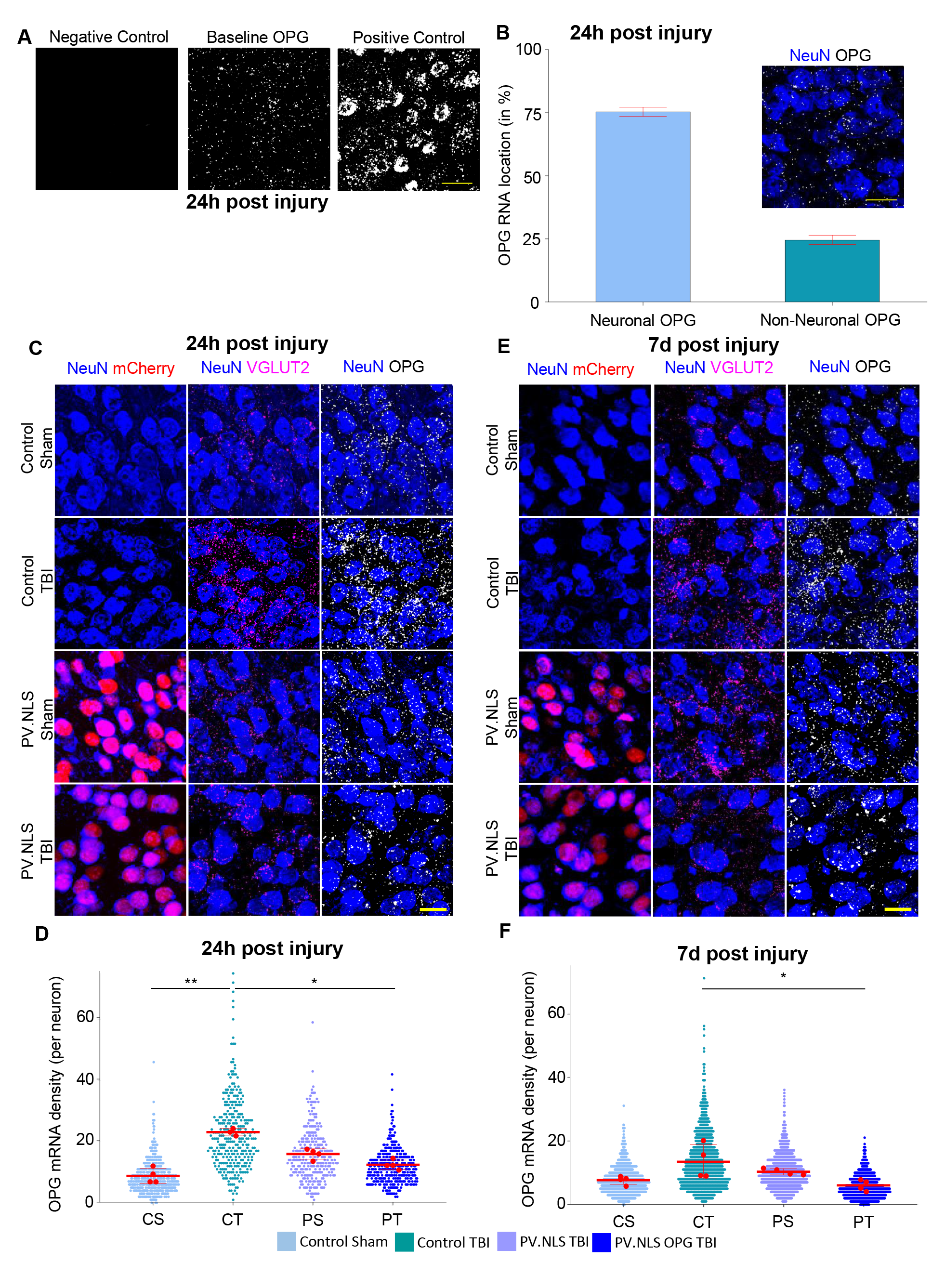

Supplement: Supplementary file 4 — Additional file 4: Figure S4. Neuronal expression of osteoprotegerin is upregulated by nuclear calcium signaling and neuronal activity in TBI. A) Negative Control, baseline OPG (TNFRSF11B) signal and a positive Control of the in situ hybridization 24h post injury. B) OPG (TNFRSF11B) in situ signal is mainly found in neuronal sources (neuronal vs non-neuronal 75.38 ± 1.83% vs 24.62% ± 1.83%). N=5. C)-D) Significant increase of OPG (TNFRSF11b) mRNA density 24h post-TBI compared to sham (CS vs CT; 8.53 ± 2.45 vs 28.74 ± 12.03). Buffering of nuclear calcium in TBI significantly decreased OPG mRNA density (CT vs PT; 28.74 ± 12.03 vs 12.14 ± 1.58). Small datapoints depict individual neurons, red datapoints depict average per animal mean ± SD. N=4 (used for statistics). Scale bar: 20µm. E)-F) Buffering of nuclear calcium in TBI significantly decreased OPG mRNA density (CT vs PT; 13.46 ± 5.41 vs 6.03 ± 1.75).mall datapoints depict individual neurons, red datapoints depict average per animal mean ± SD. N=4 (used for statistics). *: p < 0.05; **: p < 0.01. Scale bar: 20µm. [file 12974_2022_2634_MOESM4_ESM.tif]

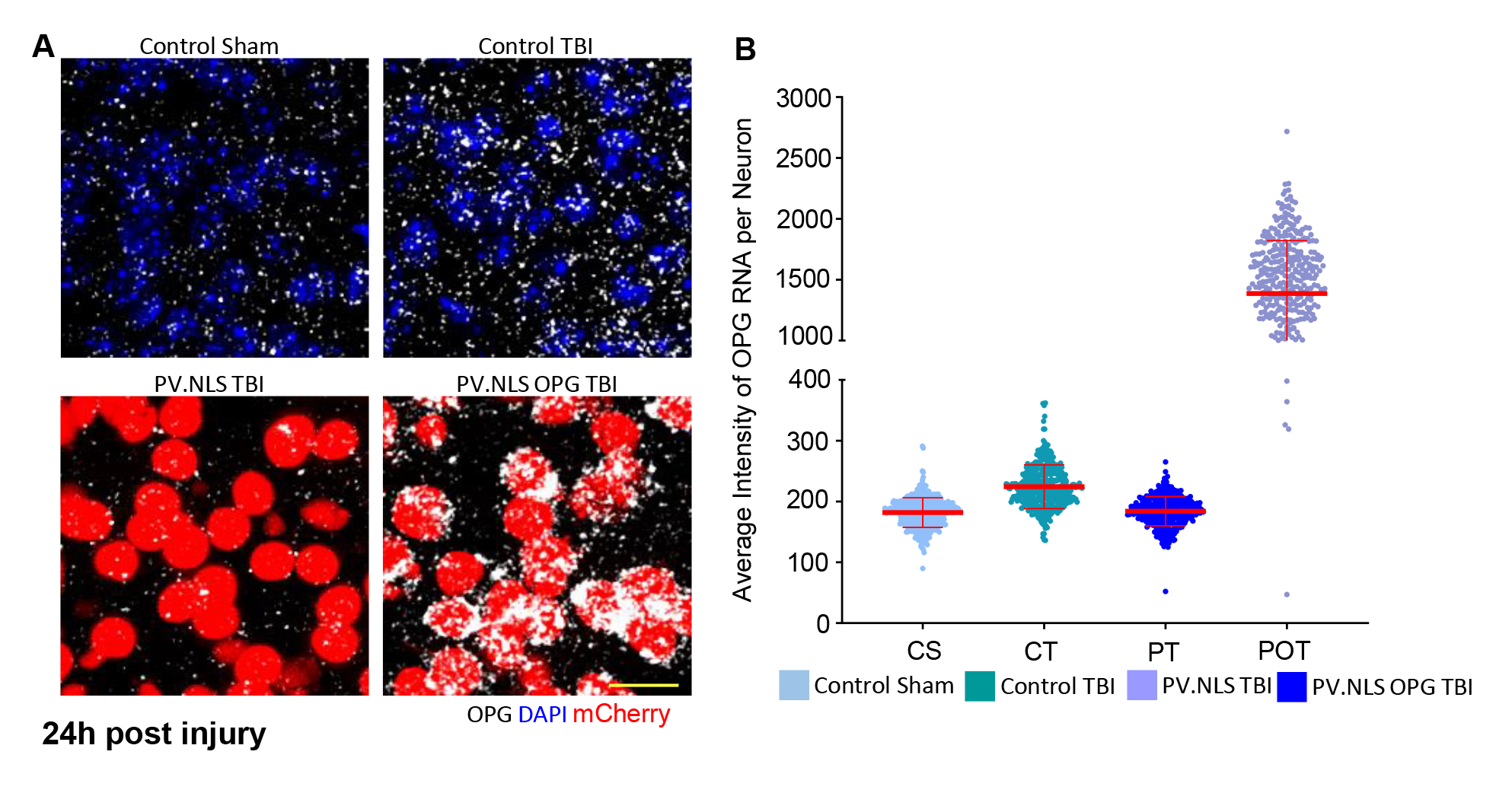

Supplement: Supplementary file 5 — Additional file 5: Figure S5. Re-expression of osteoprotegerin together with nuclear calcium buffering massively induces neuronal OPG mRNA intensity. A)-B) Significant increase in neuronal OPG (TNFRSF11b) mRNA intensity (CS vs CT; 181.8 ± 24.34 vs 224.3 ± 35.87). Buffering of nuclear calcium signaling decreased neuronal OPG mRNA intensity (CT vs PT; 224.3 ± 35.87 vs 184.1 ± 24.11). Re-expression of OPG together with nuclear calcium buffering increased the neuronal OPG mRNA intensity massively (PT vs POT; 184.1 ± 24.11 vs 1386 ± 435). Each dot represents one neuron. N=4. Scale bar overview: 20µm. [file 12974_2022_2634_MOESM5_ESM.tif]
